# *Trypanosoma brucei* infection remodels the uterine immune environment and drives neuroendocrine dysfunction

**DOI:** 10.1371/journal.ppat.1014667

**Published:** 2026-10-09

**Authors:** Olivia M. Shorthouse, Chloe Barnes, Stefano A. P. Colombo, Jessica Costa, Katrine Wonsbek, Anmoyul Mohon, Andrew S. MacDonald, Elizabeth Mann, Alice H. Costain, Juan F. Quintana

**Affiliations:** 1 Lydia Becker Institute of Immunology and Inflammation, Faculty of Biology, Medicine and Health, University of Manchester, Manchester, United Kingdom; 2 Division of Integrative Physiology, School of Medical Sciences, Faculty of Biology, Medicine and Health, Manchester Academic Health Science Centre, University of Manchester, Manchester, United Kingdom; 3 Maternal and Foetal Health Research Centre, Division of Developmental Biology and Medicine, University of Manchester, St. Mary’s Hospital, Manchester, United Kingdom; 4 Geoffrey Jefferson Brain Research Centre, University of Manchester, Manchester, United Kingdom; 5 Institute of Immunology and Infection Research, School of Biological Sciences, University of Edinburgh, Edinburgh, United Kingdom; University of Notre Dame, UNITED STATES OF AMERICA

## Abstract

Human African Trypanosomiasis (HAT) or sleeping sickness is a systemic parasitic infection caused by the protozoan parasite *Trypanosoma brucei.* HAT is associated with substantial immunological, metabolic, and neurological pathology. Although reproductive dysfunction has previously been recognised in both human and experimental *T. brucei* infection, whether parasites can directly infiltrate the female reproductive tract (FRT), and how infection may reshape the FRT immune landscape remains poorly understood. Using a murine model of *T. brucei* infection we reveal that parasites are localised in the uterine lining (endometrium) during both acute and chronic infection stages in mice. Chronic *T. brucei* infection was associated with progressive fat wasting, disruption of the reproductive (oestrous) cycle, uterine and ovarian atrophy, and extensive transcriptional dysregulation across the hypothalamic-pituitary-gonadal (HPG) axis. Acute and chronic infection induced remodelling of the uterine immune landscape, characterised by T cell infiltration, pro-inflammatory myeloid activation, alongside broader type 1 inflammatory changes across reproductive tissues and HPG components. Ovarian pathology was accompanied by follicular degeneration, a reduction in corpora lutea and alterations to steroidogenic pathways. Hormonal rescue with the selective oestrogen receptor modulator, tamoxifen, restored uterine morphology and prevented oestrous cycle arrest, but did not reverse the infection-induced uterine immune remodelling, indicating that endocrine dysfunction and infection-driven inflammation are distinct processes. Taken together, these findings identify the FRT as a major target of *T. brucei* infection and demonstrate how chronic parasitic infection can disrupt reproductive physiology through a combination of immune, endocrine, and metabolic pathways. They also highlight the need to specifically assess the FRT in other models of systemic inflammation.

## Author summary

Human and animal African trypanosomiasis, also known as sleeping sickness and nagana, are caused by the parasite, *Trypanosoma brucei*. These chronic infections are associated with immune changes across the body as well as changes to metabolism and neurology. In both humans and animals, infection has been linked to poor reproductive outcomes, including miscarriage, foetal growth restriction and menstrual irregularities. However, whether *T. brucei* can infiltrate into the uterus of infected mice and whether this presence can alter the local immune cell dynamics remains poorly understood. Using an animal model of acute and chronic *T. brucei* infection, we were able to detect the parasites within the uterus of infected female mice. In addition, we found that the immune cell profile from the uterus of infected females was more pro-inflammatory during *T. brucei* infection. During chronic infection, we found that animals showed progressive fat wastage, disruption of reproductive cycling, and marked uterine and ovarian shrinkage. When we administered an oestrogen-like compound, we found that uterine and ovarian size changes were hormone-dependent but the immune changes in the uterus were hormone-independent.

## Introduction

The extracellular protozoan parasite *Trypanosoma brucei* is the causative agent behind human African trypanosomiasis (HAT; sleeping sickness) and animal African trypanosomiasis (AAT; nagana) [1,2]. These diseases are endemic to sub-Saharan Africa, where they are responsible for considerable socio-economic burden [3,4]. Transmission occurs following a bite of an infected tsetse fly, after which parasites are released into the bloodstream, proliferate and invade multiple tissues throughout the host [5]. Both HAT and AAT are chronic inflammatory infections characterised by waves of parasitaemia, sustained immune activation, and progressive host wasting [5]. Extensive work using animal models of *T. brucei brucei* infection have defined how this parasite remodels immune responses and metabolic processes across multiple tissues, including the brain [6–8], skin [9,10], adipose tissue [11–13], and testes [14,15]. Moreover, the related subspecies *T. b. gambiense*, that causes chronic HAT, has previously been detected in the murine upper female reproductive tract (FRT), comprising the uterus and ovaries, even in the absence of detectable parasitaemia in the blood [16]. However, whether infection with *T. b. brucei* similarly invades the FRT, and the consequences of infection on the local environment, remains unexplored.

The FRT environment contains a highly dynamic immune network that supports tissue homeostasis, cyclic remodelling and pathogen defence throughout reproductive cycles to facilitate reproductive success [17]. Immune cell populations within the uterus fluctuate throughout reproductive cycles and across reproductive life stages, from puberty to post-menopause, in response to hormonal cues [18,19]. Although systemic inflammatory states have been shown to alter uterine immune homeostasis [20,21], much of this evidence is derived from reductionist lipopolysaccharide (LPS) models or non-communicable inflammatory conditions such as obesity [22,23]. Whether systemic parasitic infections similarly remodel the uterine immune landscape has received limited investigation, this is particularly pertinent since neglected tropical diseases (NTDs) disproportionately affect women and girls [24]. In humans and livestock, *T. brucei* infection drives marked cachexia and systemic inflammation [25,26], conditions known to profoundly influence reproductive health [27,28]. Reproductive function is highly sensitive to systemic inflammatory and metabolic stress [27,29]. Indeed, reproductive dysfunction is frequently reported during African trypanosomiasis [30,31]. In HAT, infection has been associated with infertility, menstrual irregularities, amenorrhea and reduced circulating sex hormones in both men and women. AAT is linked to dysregulated oestrous cycling, abortion, foetal loss, orchitis, and scrotal dermatitis [14,32–35].

Reproductive function is governed by the hypothalamic-pituitary-gonadal (HPG) axis, a neuroendocrine network that integrates systemic cues to regulate hormone production and menstrual/oestrous cycling [36]. The HPG axis is highly sensitive to inflammatory and metabolic perturbation, both of which are found in HAT and AAT where infections are characterised by a sustained type 1 inflammatory response [37,38]. Use of experimental animal models have additionally demonstrated infection-associated hypogonadism and disruption of HPG axis function [39,40]. Despite these observations, the FRT has remained comparatively understudied as a site of infection-associated pathology and whether direct parasite invasion and/or local immune remodelling contributes to reproductive dysfunction remains unexplored. Here, we use a murine model of *T. b. brucei* infection to assess the impact of acute and chronic stages on the FRT. We demonstrated that *T. b. brucei* parasites accumulate within the uterus during acute and chronic infection, with pronounced uterine and ovarian atrophy during chronic infection. Immunophenotyping revealed that both infection stages altered the immune composition of the uterus and its associated draining lymph node, promoting a type 1 inflammatory environment. Functionally, chronic infection led to profound disruption of oestrous cyclicity and circulating sex steroid levels, coinciding with transcriptional evidence of HPG axis dysregulation. Using pharmacological modulation of oestrogen receptor signalling, we disentangled the endocrine and immune contributions to the FRT pathology. Tamoxifen treatment rescued the uterine and ovarian atrophy, but the infection-induced uterine immune remodelling persisted independently of treatment. Collectively, our findings identify the FRT as a target site of systemic *T. brucei* infection and demonstrate how chronic infection disrupts the tightly coordinated immune and endocrine pathways required to maintain reproductive homeostasis.

## Results

### T. *brucei* accumulates in the murine uterus and drives uterine and ovarian atrophy during the chronic phase of infection

Although *T. brucei brucei* is the most commonly used subspecies in rodent models, whether *T. b. brucei* can invade the FRT has not been researched. To address this knowledge gap, we infected female C57BL/6 mice and analysed the uterine tissue at day 7 and days 21–24 to represent acute and chronic stages of infection, respectively (Fig 1A) [13,41]. Histological analysis using staining for the *T.* brucei-specific protein Binding Immunoglobulin Protein (BiP) showed no detectable staining in naive uterine tissue, whereas parasites were readily detected in the uterus during T. brucei infection (Fig 1B–1D). Parasites were found mainly in the inner uterine lining (endometrium) with a sparse number of parasites detected within the outer layer of the uterus (myometrium) (Fig 1B–1D and S1A Fig). Despite comparable histological detection of *T. brucei* parasites and blood parasitaemia levels at both time points, qPCR quantification against the trypanosome-specific *Pfr2* gene showed significantly higher parasite burden in the uterus during chronic infection compared with the acute stage (Fig 1D and 1E). To determine whether the *Pfr2* signal could originate from parasites within the uterine vasculature, parasite burden was quantified in uterine tissue from perfused animals. *Pfr2* was readily detected in uterine tissue from perfused chronically infected mice (S1B Fig).

**Fig 1:**
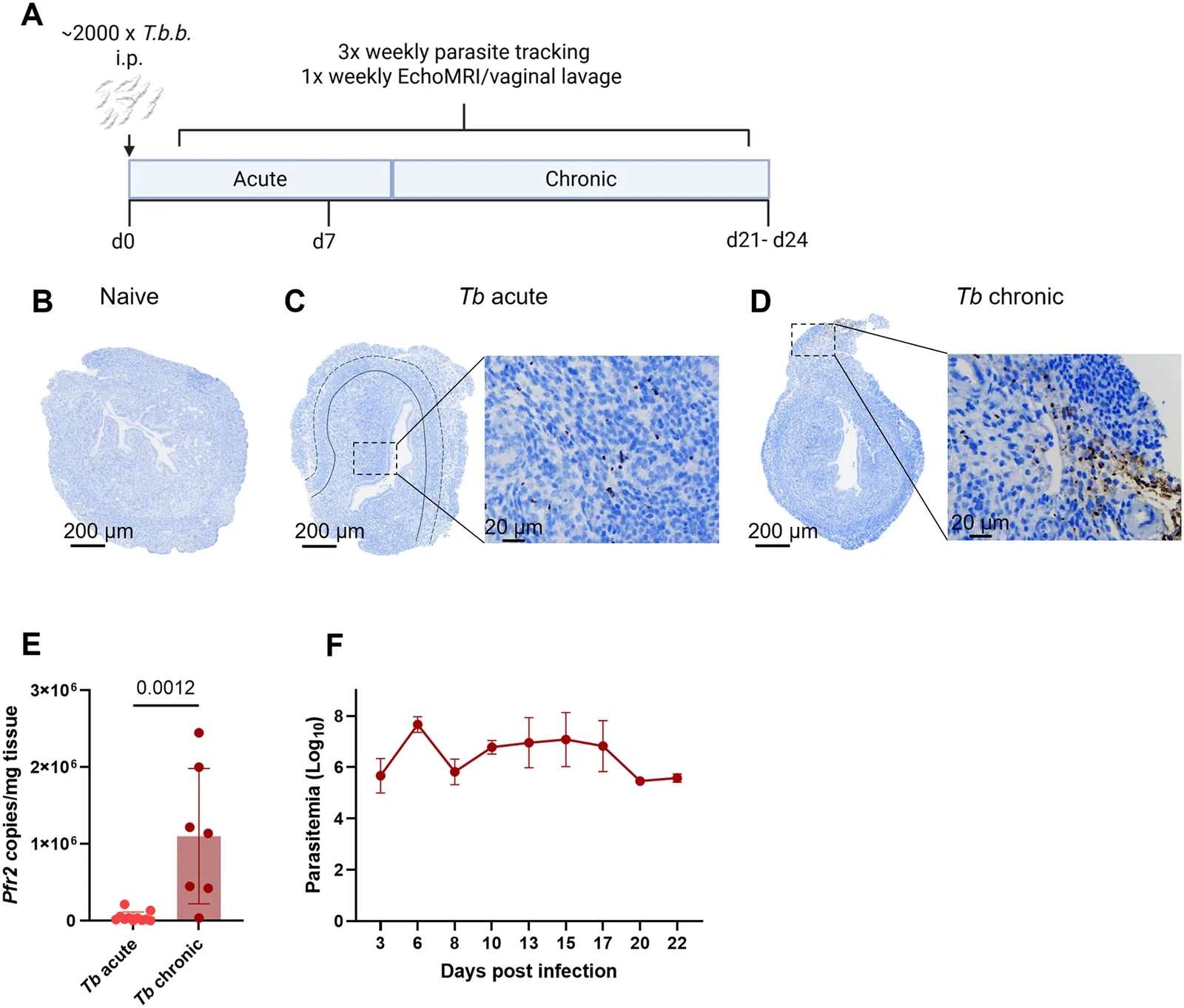
*T. brucei* accumulates in the uterus. (A) Overview of the experimental approach. C57BL/6 female mice were infected with ~2000 *T. b. brucei* parasites intraperitoneally and culled at days 7 and 21–24 to represent acute and chronic stages, respectively. Representative image of immunohistochemistry staining the *T*. *brucei*-specific protein BiP in uterine cross section from (B) naive, (C) acute and (D) chronically infected females (scale bar = 200 µm; scale bar zoom = 20 µm; solid line separate endometrium and myometrium, dashed line separates inner and outer myometrium). (E) Estimation of *T. brucei* burden in the uterus using qPCR analysis to detect parasite-specific *Pfr2* gene in acutely- and chronically infected females. (F) Number of parasites per ml of blood from C57BL/6 female mice infected with ~2000 *T. b. brucei* parasites. Parasitaemia was measured using phase microscopy and the rapid ‘matching’ method. Data combined from 2-3 independent experiments with mean ± SEM shown. Statistical analysis was performed using Mann-Whitney test (E). BiP = Binding-Immunoglobulin protein. The supporting data for Fig 1D and 1E can be found in S1 Data in S1 File.

We next assessed the impact of *T. brucei* infection on uterine structure. Infection resulted in progressive atrophy through the acute and chronic stages (Fig 2A). When uterine weight as a proportion of body weight (uterine index) [42] was quantified, we observed significant reductions at both stages with the greatest reduction at the chronic stage (Fig 2B). Ovary weight was also significantly reduced in the chronically infected animals but not at the acute stage (Fig 2C). Macroscopically, the uteri from chronically infected females appeared less vascularised than those from acutely infected and naive females, with narrow uterine horns (Fig 2A). Histologically, both the luminal area and endometrial area were significantly reduced in infected females (Fig 2D–2F), whereas myometrial thickness was unaffected by infection (S2A and S2B Fig), consistent with the differential plasticity of uterine compartments [43]. Within the endometrium, chronically infected mice exhibited a reduction in gland number (Fig 2G), which may impair the secretory support required for blastocyst implantation [44]. In addition, luminal epithelial height was significantly reduced in chronically infected females (Fig 2H), demonstrating that *T. brucei* infection induces structural remodelling of the uterine epithelium. Although body weight remained unchanged throughout the course of the experiment (S3A Fig), MRI-based body composition analysis enabled longitudinal quantification of body fat and lean mass. From day 14 post-infection onwards, infected animals exhibited significantly reduced body fat percentage compared to naive controls (Fig 2I), while lean mass remained unchanged (S3B Fig). The absence of an overall body weight difference between naive and infected animals but reduced body fat percentage was likely attributable to pronounced splenomegaly and hepatomegaly in infected animals (S3C and S3D Fig). In line with these MRI findings and previous reports on other adipose tissue sites [11], during chronic infection, mice had significantly less parametrial adipose tissue associated with the FRT relative to total body weight than naive animals (Fig 2J).

**Fig 2:**
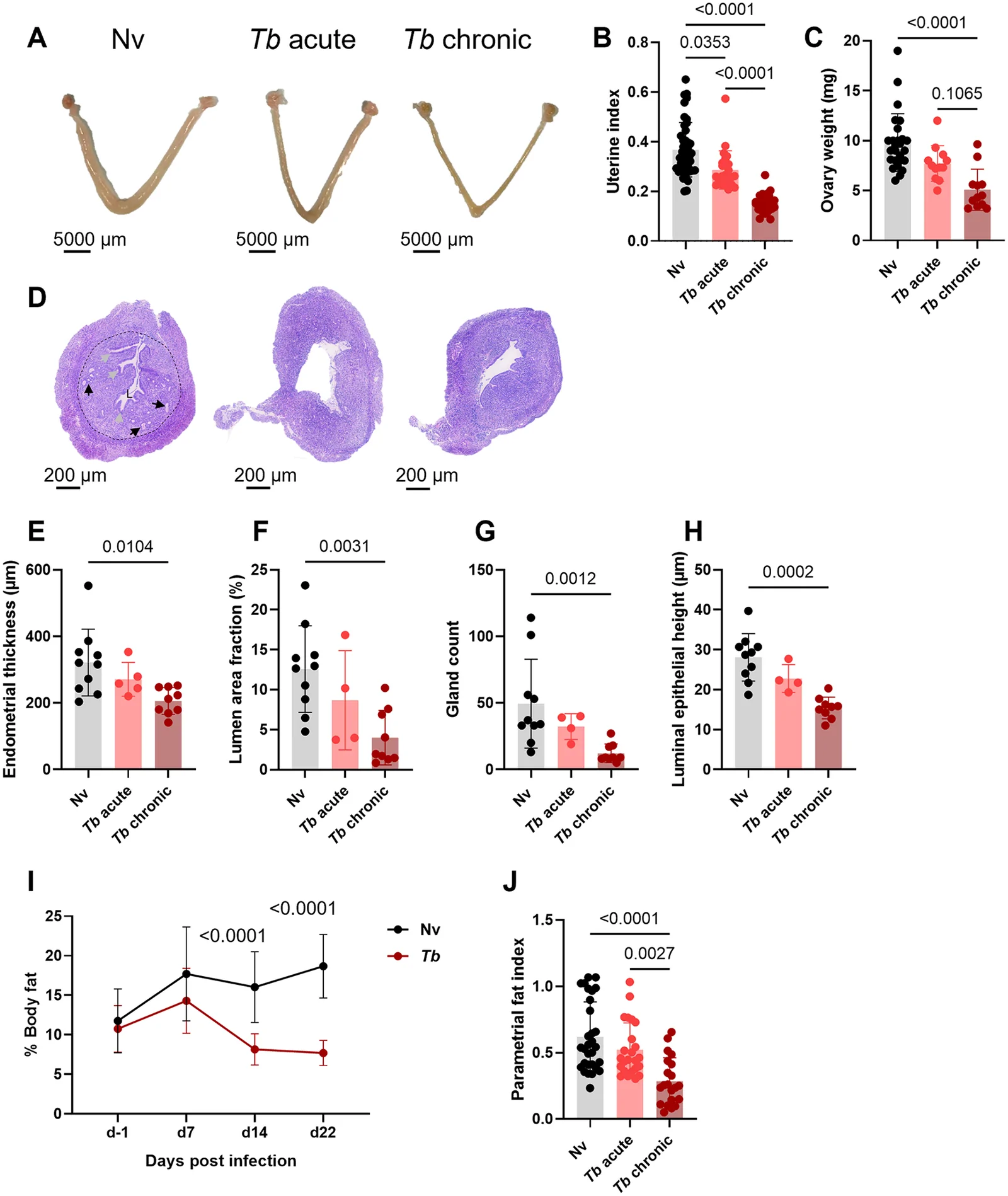
Uterine and ovarian atrophy develop during *T. brucei* infection. (A) Representative images of the gross morphology of uteri and ovaries from naive (Nv), *T. brucei* acute infection (*Tb* acute) and *T. brucei* chronic infection (*Tb* chronic). Scale bar = 5000 µm. (B) Uterine weights as a proportion of body weight. (C) Combined ovary weight. (D) Representative H&E-stained uterine cross sections from Nv, *Tb* acute and chronic conditions. L: lumen, black arrows indicate endometrial glands, and grey arrows indicate endometrial invaginations, dashed line separates the myometrium from the inner endometrium. Scale bar = 200 µm. Quantification of H&E-stained uterine cross-sections, quantifying (E) endometrial thickness, (F) lumen area fraction, (G) gland count and (H) luminal epithelial height. (I) Longitudinal analysis of total body fat in naive and infected animals from pre-infection to the chronic stage of infection by MRI analysis. (J) Parametrial fat, the adipose tissue surrounding the FRT, weight as a proportion of body weight. Data combined from 3-5 independent experiments with mean ± SEM shown. Statistical analysis was performed using Kruskal-Wallis with Dunn’s *post-hoc* multiple comparisons (C, E, G-H, J), one-way ANOVA (F) or two-way ANOVA with multiple comparisons (I). The supporting data for Fig 2B, C–J can be found in S2 Data in S1 File.

### Acute and chronic *T. brucei* infection reprogrammes the uterine immune landscape

Given the marked structural changes in the uterus following *T. brucei* infection, we next characterised inflammatory status by assessing the uterine, and its associated draining lymph nodes (udLN), immune cell profiles using flow cytometry (S4 Fig). The frequency of CD45^+^ cells in the uterus was significantly increased during chronic but not acute infection (Fig 3A), although CD45^+^ cell numbers per milligram of tissue was unaffected (S5A Fig). Quantification of major immune subsets revealed distinct temporal dynamics across infection stages (Fig 3B). Eosinophils (CD45^+^SiglecF^+^SSC-high) were almost entirely absent at both acute and chronic time points (Fig 3B). NK cell frequencies, assessed in naive and chronically infected animals, were significantly reduced during chronic infection (S5B Fig). Monocytes (SiglecF^-^Ly6G^-^CD11b^+^CD64^+^Ly6C^+^) were elevated during acute but not chronic infection (Fig 3B) whereas macrophages (SiglecF^-^Ly6G^-^CD11b^+^CD64^+^Ly6C^-^) showed a trend towards increased abundance during chronic infection (Fig 3B). Despite these differences, monocytes upregulated the expression of Ym1 at both time points, a chitinase-like protein associated with alternative activation [45] (S5C Fig). In contrast, macrophages and conventional DCs (cDCs; SiglecF^-^Ly6G^-^CD64^-^MHC-II^+^CD11c^+^) adopted a pro-inflammatory phenotype, evidenced by an increase in pro-inflammatory macrophages (CD11c^+^CD206^-^), and an elevated proportion of cDC1s (XCR1^+^CD11b^-^) [46] at both time points (S5D and S5E Fig). To specifically assess tissue uterine T cell responses, we performed *in vivo* intravascular labelling using CD45-BV785 antibody before tissue processing, allowing for discrimination between CD45-BUV805^+^ uterine tissue cells and CD45-BV785^+^ vascular cells (S6A Fig). Within the uterine tissue compartment (CD45-BUV805^+^), T cell frequencies increased during infection, with the greatest increase during chronic infection (Fig 3B and S5F Fig). This was supported by immunohistochemical staining for CD3 in uterine sections, which confirmed CD3^+^ T cell expansion (Fig 3C, 3D and S5J Fig). Furthermore, uterine T cells expressed significantly elevated levels of the cell cycle marker Ki67 indicative of active local proliferation (Fig 3E and 3G) with a more marked increase during acute infection.

**Fig 3:**
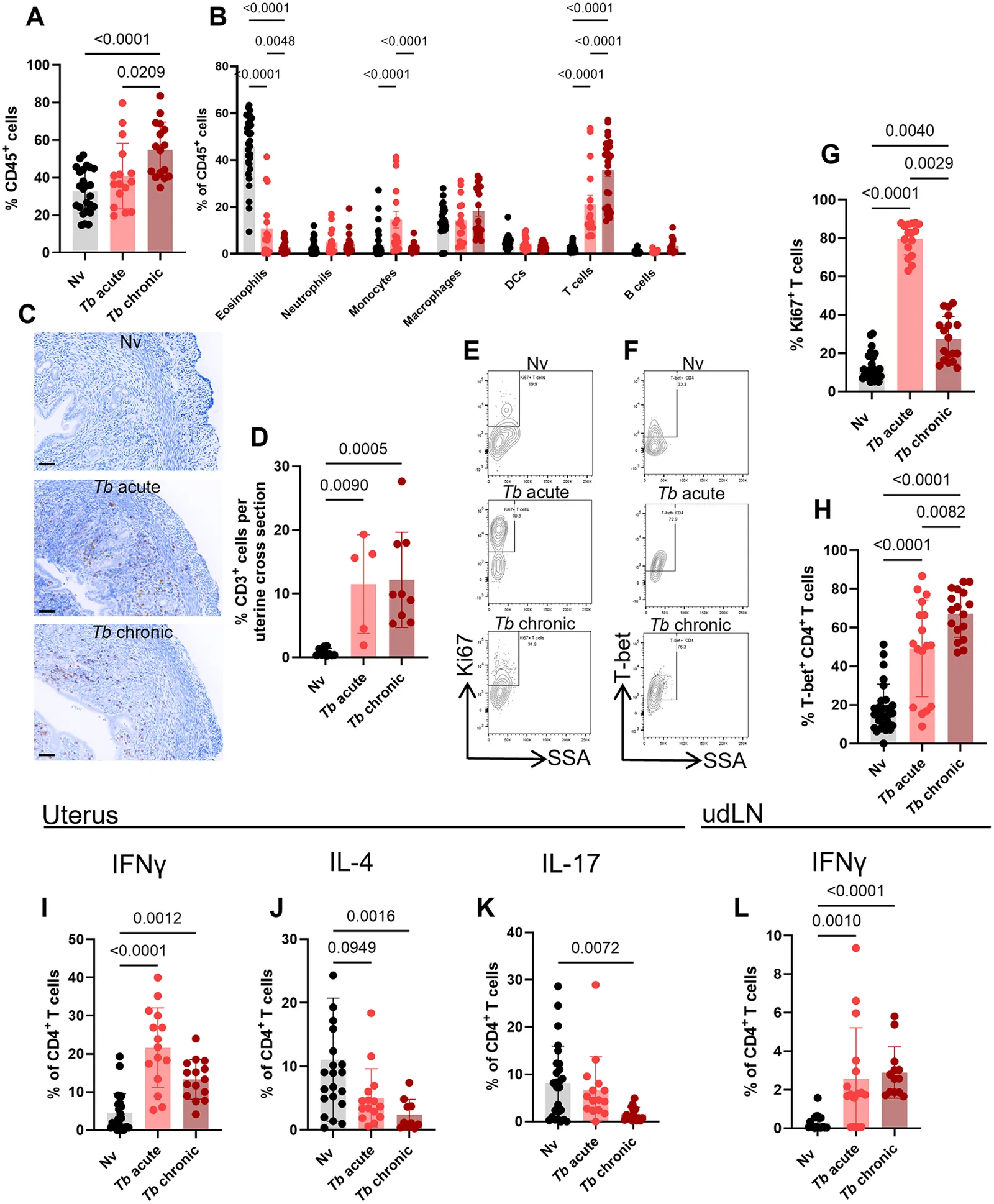
At acute and chronic infection stages, *T. brucei* drives type 1 skewing of the uterine immune landscape. Flow cytometry and histological analysis of uterine and uterine draining lymph node immune cell populations from naive (black), *T. brucei* acute infected (light red) and *T. brucei* chronic infected (burgundy) animals. (A) Proportion of uterine CD45^+^ cells. (B) Proportions of major immune cell populations as a proportion of CD45^+^ cells. (C) Representative uterine cross sections of CD3^+^ DAB staining across Nv, *Tb* acute and *Tb* chronic conditions (scale bar = 50 µm). (D) Quantification of CD3^+^ staining. (E) Representative flow cytometry plots of Ki67 expression in T cells across conditions as a percentage of total T cells. (F) Representative flow cytometry plots of T-bet expression by CD4^+^ T cells as a percentage of CD4^+^ T cells. (G) Quantification of (E). (H) Quantification of (F). Cytokine production (I) IFNγ, (J) IL-4, and (K) IL-17 by CD4^+^ uterine T cells following PMA/ionomycin stimulation. (L) IFNγ production by uterine draining lymph node (udLN) CD4^+^ T cells following PMA/ionomycin stimulation. Data combined from 4-5 independent experiments with mean ± SEM shown. Statistical analysis was performed using one-way ANOVA (A, H), Kruskal-Wallis with Dunn’s *post-hoc* multiple comparisons (D, G, I-L) or two-way ANOVA with multiple comparisons (B). The supporting data for Fig 3A–C, E–L can be found in S3 Data in S1 File.

Given the well-characterised Th1 skewing associated with *T. brucei* infection at other anatomical sites [9,38,47], we next profiled the uterine T cell populations by flow cytometry. We confirmed Th1 skewing in the uterus as shown by CD4^+^ T cells exhibiting significantly higher levels of the Th1 transcription factor T-bet (Fig 3F and 3H), and *ex vivo* stimulation with PMA/ionomycin demonstrated enhanced IFNγ production by uterine CD4 ⁺ T cells at both time points (Fig 3I). Alongside this, transcription factors associated with Th2 and Th17 lineages (GATA3 and RORgt, respectively) were downregulated (S5G and S5H Fig) and *ex vivo* stimulation with PMA/ionomycin showed downregulated secretion of the type 2 and 17 cytokines, IL-4, IL-13 and IL-17 (Fig 3J, 3K and S5I Fig). Associated to these local responses, uterine-draining lymph nodes (udLNs) also exhibited enhanced IFNγ production (Fig 3L).

Having established substantial uterine T cell remodelling, we next examined the uterine vasculature compartment (CD45-BV785^+^) separately. This approach revealed that the expansion and skewing of the T cell compartment towards a type 1 landscape was also evident in the uterine vasculature (S6B Fig). Notably, the uterine vasculature response was temporally dynamic, with CD8 ⁺ T cells predominating at the acute stage, before shifting to a CD4 ⁺ T cell dominated response at the chronic stage, mirroring the changes in the uterine tissue compartment (Fig 3C, S5I Fig and S6C Fig). These data demonstrate that both acute and chronic *T. brucei* infection drive a pronounced shift toward a type 1 immune environment in the uterus, characterised by expansion of Th1 cells and coordinated increases in type 1 cytokine production across the uterus and its draining lymph nodes.

### Chronic *T. brucei* infection drives dysfunctional reproductive cycling

Having established that *T. brucei* infection dramatically alters the immunological profile of the uterus at the acute and chronic time points, we wanted to investigate the implications for reproductive (oestrous) cycling and ovarian function. To determine which oestrous stage mice were in, we collected vaginal lavage (VAL) fluid and performed qualitative assessment of VAL cellularity by H&E staining (S7A Fig) prior to and throughout infection. The reproductive cycle of female mice consists of four stages: proestrus, oestrus, metestrus, and dioestrus (S7A Fig) [48]. In naive animals, the distribution of oestrous stages was relatively even, although a slightly higher proportion of animals were in oestrus rather than dioestrus, the longest and therefore more commonly observed stage. Acutely infected animals had a significantly altered oestrous cycle, whereas chronically infected animals exhibited a severely disrupted oestrous cycle, with the majority of animals presenting in the proestrus stage, suggestive of acyclicity (Fig 4A). This skewing of the oestrous cycle towards proestrus was apparent from day 14 post-infection and became more skewed as the infection progressed (Fig 4B). Given the uterine and ovarian atrophy and the dysregulated oestrous cycling observed during chronic infection, we next sought to understand whether these changes were accompanied by alterations in ovarian follicle maturation. Quantification of ovarian follicle populations across developmental stages revealed no differences between naive and chronically infected animals (Fig 4D). However, chronic *T. brucei* infection was associated with a marked increase in follicular degeneration (Fig 4E). Consistent with this, chronically infected animals exhibited a significant reduction in corpora lutea number and overall corpora lutea output (Fig 4F and S7B Fig), consistent with reduced ovulatory activity and impaired follicle maturation. Given the disruption to oestrous cycling, we next profiled the systemic endocrinological environment which revealed that chronic *T. brucei* infection was associated with a marked reduction in circulating progesterone, while oestradiol levels were increased relative to naive controls (Fig 4G and 4H). In contrast, levels of luteinising hormone (LH) and follicle-stimulating hormone (FSH) remained unchanged between groups (S7C and S7D Fig). These findings indicate impaired ovarian function during chronic infection, characterised by increased follicular loss, reduced corpora lutea formation and altered circulating sex steroid levels.

**Fig 4:**
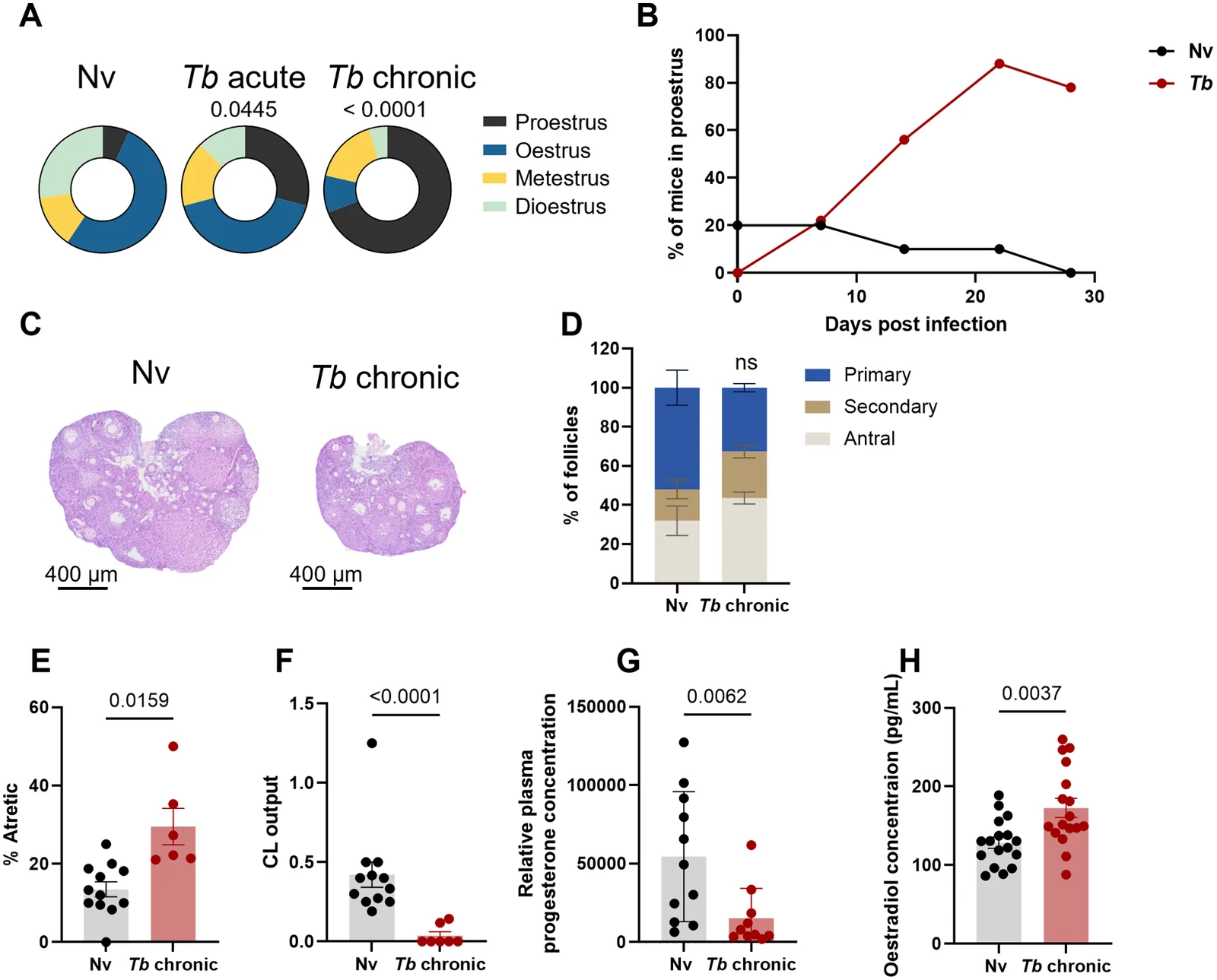
Chronic *T. brucei* infection drives hypogonadism. (A) Distribution of oestrous cycle stages (proestrus, oestrus, metestrus, dioestrus) in Nv, *Tb* acute and *Tb* chronic animals. (B) Longitudinal oestrous stage tracking showing the percentage of naive and *Tb* infected animals in the proestrus stage. (C) Representative mouse ovarian histological sections from naive and *T. brucei*-infected female mice, (scale bar = 400 μm). (D) Percentage of primary, secondary and antral follicle counts in ovaries from naive and *Tb* chronically infected animals. (E) Percentage of atretic follicles. (F) Corpus luteum output: number of corpora lutea as a proportion of total healthy follicles. (G) Relative quantification of circulating progesterone in plasma from naive and chronic *T. brucei*-infected mice. (H) Levels of circulating oestradiol from naive and chronic *T. brucei*-infected mice. Data combined from 2-5 independent experiments with mean ± SEM shown. Statistical analysis was performed using Fisher’s exact test (A), two-way ANOVA (B, D), Welch’s t-test (E), Mann-Whitney test (F, G) or unpaired t-test (H). The supporting data for Fig 4A, B, D–H and can be found in S4 Data in S1 File.

### T. *brucei* infection transcriptionally impacts the hypothalamic-pituitary-gonadal (HPG) axis

Having established that chronic *T. brucei* infection disrupts oestrous cycling and ovarian function, we reasoned that the hypothalamic-pituitary-gonadal (HPG) axis, the master regulator of vertebrate reproduction [36], may also be altered. To assess this, we utilised bulk RNAseq of the hypothalamus, pituitary gland, and ovaries from naive, acutely infected, and chronically infected animals, providing an overview of the transcriptional changes across the HPG axis components in response to infection. Across both infection stages, when compared to naive controls, we observed broad differential gene expression across all components of the HPG axis, with large numbers of significantly up- and downregulated genes indicating systemic transcriptional dysregulation during infection. Principal component analysis demonstrated clear separation by tissue type along PC1 and PC2 (Fig 5A), while PC3 resolved infection status, revealing a stepwise transcriptional shift from acute to chronic *T. brucei* infection (Fig 5B).

**Fig 5:**
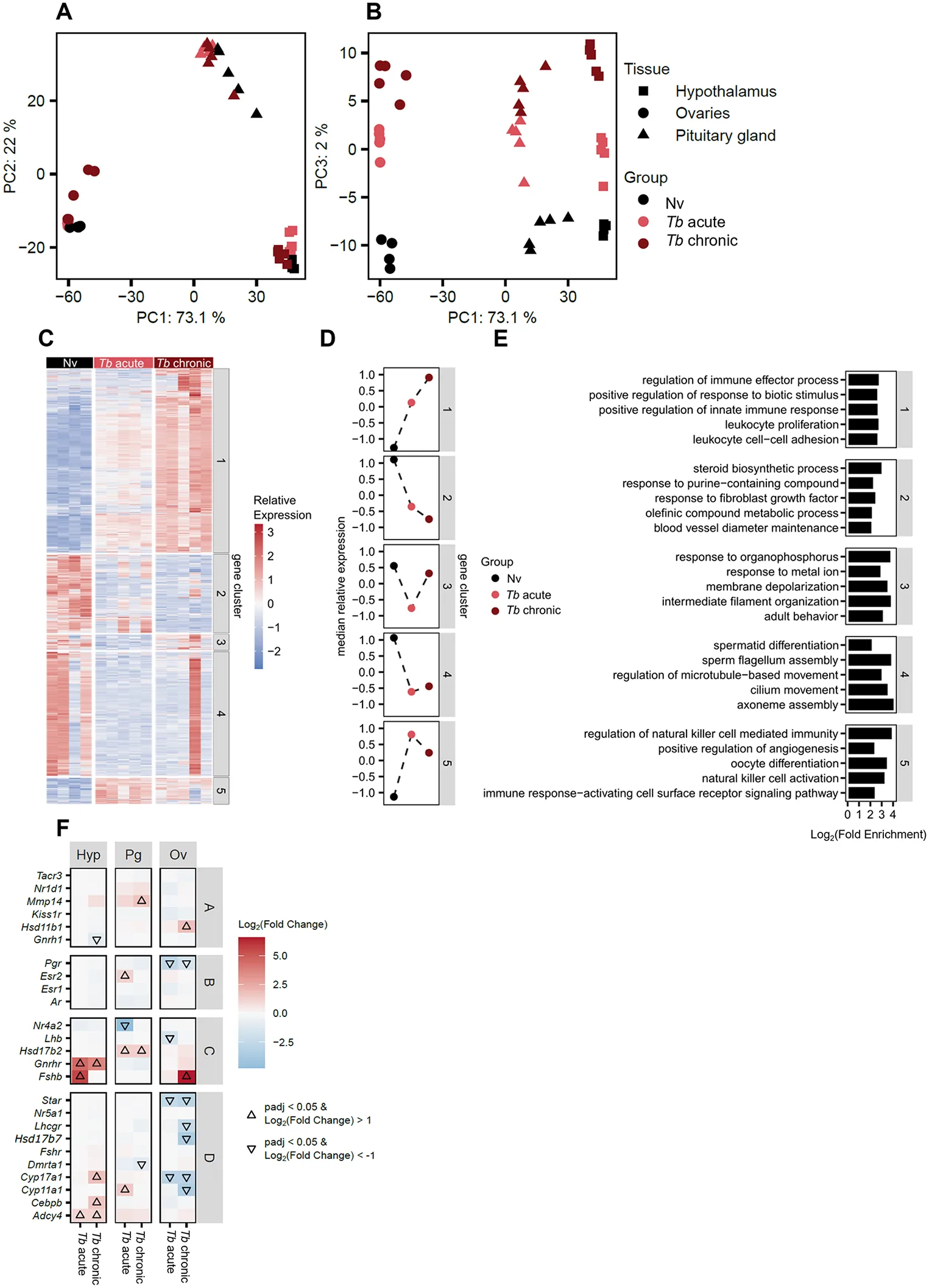
*T. brucei* infection drives significant transcriptional changes to the Hypothalamic-pituitary-gonadal (HPG) axis. Hypothalamus, pituitary gland and ovaries from naive, *T. brucei* acute and chronically infected mice were collected and RNA was profiled by bulk RNAseq. Principal components analysis of total read counts using PC1 x PC2 (A) and PC1 x PC3 (B). Each point represents one animal. (C) Heat map representing the relative expression of ovarian genes identified as statistically differentially expressed in at least one comparison. Genes were grouped into clusters by K-means clustering. (D) A summary of the median scaled expression of all genes from each cluster across Nv, *Tb* acute- and *Tb* chronic infections. (E) Gene set enrichment analysis of gene clusters from (C) using GO Biological Process terms. (F) Heatmap of genes associated with HPG endocrine function across hypothalamus (Hyp), pituitary gland (Pg), and ovaries (Ov), shown as differential expression relative to naive controls; arrows denote statistically significant changes.

Given the evidence of ovarian dysfunction (Fig 4E and 4F), we next focused on ovarian transcriptional changes. Hierarchical clustering followed by k-means clustering of significantly differentially expressed genes identified five gene clusters with distinct expression profiles between groups (Fig 5C). Clusters one and five, representing genes enriched for immune-associated processes, including leukocyte proliferation, displayed upregulation in both acute and chronic infection, with a stepwise increase most evident in cluster one relative to naive controls (Fig 5D and 5E). Similar immune activation pathways were enriched in the hypothalamus and pituitary glands (S8A and S8B Fig) indicating systemic inflammatory responses across the HPG axis components during infection. In contrast, clusters two and four representing genes enriched for biological processes related to reproductive function, including microtubule-based movement, axoneme assembly, and sperm flagellum assembly were downregulated across both infection stages. This likely reflects disrupted cellular motility programmes linked to follicular maturation and germ cell function. Additional downregulated pathways included steroid biosynthetic process, growth factor signalling, and blood vessel diameter, which declined in a stepwise manner with infection progression (Fig 5D and 5E). Together, these changes are consistent with ovarian dysfunction and align with the observed increase in follicular atresia and reduction in corpora lutea (Fig 4C and 4F).

To further interrogate disruption of canonical reproductive signalling, we assessed genes involved in HPG axis function (Fig 5F). During acute infection in the hypothalamus, gonadotropin-releasing hormone receptor, *Gnrhr,* was significantly upregulated, suggesting compensatory mechanisms to altered gonadotrophin signalling, the communication pathways that govern reproductive function (Fig 5F). This was associated with moderate changes in the pituitary gland, including increased oestrogen receptor beta (*Esr2)* expression (Fig 5F). In the ovary, acute infection was characterised by downregulation of genes involved in gonadotropin signalling and steroidogenesis, including *Star* and *Cyp17a1* [49,50]. During chronic infection, this disruption became more pronounced, with marked transcriptional suppression of the HPG axis, most prominently within the ovary. While hypothalamic *Gnrhr* expression remained elevated, *Gnrh1* was downregulated alongside increased expression of inflammatory mediators including *Cebpb* indicative of sustained neuroinflammation [51,52] (Fig 5F). Ovarian transcriptional profiles showed substantial downregulation of key steroid genes, including *Star*, *Lhcgr*, *Hsd17b7*, *Cyp11a1*, and *Cyp17a1*, in line with the impairment of sex steroid hormone synthesis [49,50,53–55]. Notably, progesterone receptor (*Pgr*) was downregulated in the ovary at both infection stages, while oestrogen receptor (*Esr1*) expression remained unchanged, suggesting selective disruption of hormonal signalling. These data demonstrate a stepwise disruption of the HPG axis during *T. brucei* infection, with systemic immune activation and stage-dependent impairment of ovarian steroidogenic pathways.

### Tamoxifen treatment recovers uterine atrophy but not immunological changes

Given that uterine and ovarian atrophy occurred despite elevated circulating oestradiol, we tested whether the FRT retained the ability to respond to oestrogen receptor modulation during chronic infection. Chronically infected animals were administered tamoxifen, a selective oestrogen receptor modulator (SERM) by voluntary feeding (Fig 6A) [56]. Administration of tamoxifen reversed uterine and ovarian atrophy in chronically infected animals, with uterine horns macroscopically and histologically comparable to naive controls, exhibiting restored vascularity and no signs of atrophy (Fig 6B–6I). Notably, tamoxifen drove increased luminal epithelial growth irrespective of infection status, consistent with its known agonistic activity of the uterine oestrogen receptor (Fig 6H). These structural changes occurred independently of infection intensity, with no differences in parasitaemia, histological parasite detection, hepatomegaly or splenomegaly between treatment groups (S9 Fig), confirming that tamoxifen did not alter the systemic infection state. To determine whether oestrous cycle disruption was similarly reversible, we assessed cycle stage following tamoxifen treatment. All naive tamoxifen treated animals and over 60% of infected animals were found to be in the oestrus stage, suggesting that the FRT retained the ability to respond to oestrogen receptor modulation (Fig 6J).

**Fig 6:**
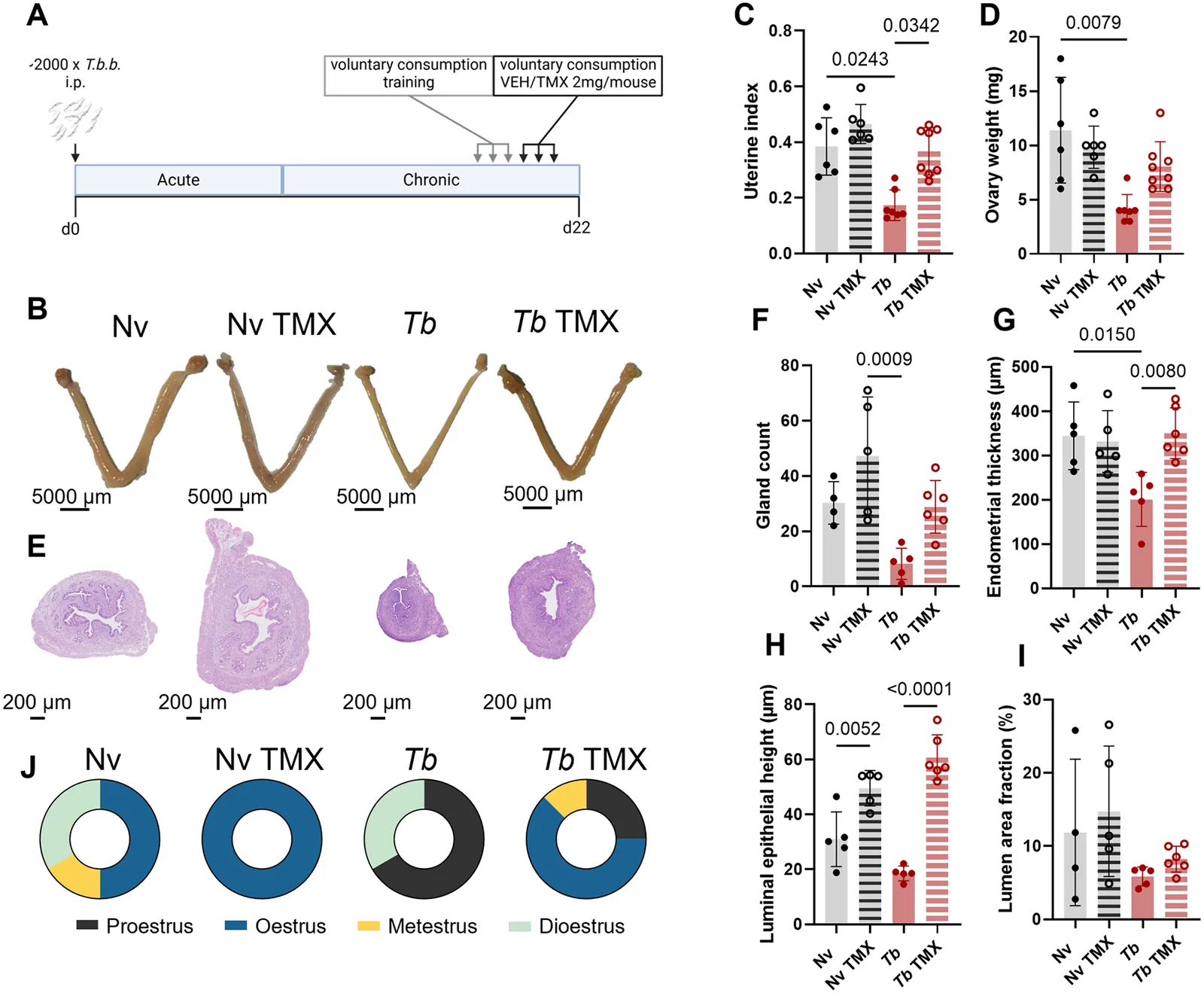
Tamoxifen treatment recovers uterine atrophy. (A) Overview of the experimental approach. C57BL/6 female mice were infected with ~2000 *T. b. brucei* parasites intraperitoneally. At days 16-18, naive and infected animals were trained to voluntarily consume vehicle, followed by 3 days of tamoxifen (TMX) in sweetened condensed milk or vehicle alone at 2 mg/mouse. Animals were culled at 22 days post-infection. (B) Representative images of the gross morphology uteri and ovaries from naive (Nv), naive treated with TMX (Nv TMX) *T. brucei* chronic infection (*Tb*) and TMX-treated *T. brucei* chronic infection (*Tb* TMX). Scale bar = 5000 µm. (C) Uterine weights as a proportion of body weight. (D) Ovary weight (mg). (E) Representative H&E-stained uterine cross sections from Nv, *Tb* chronic and TMX-treated infected conditions. Scale bar = 200 µm. Quantification of H&E-stained uterine cross-sections, quantifying (F) gland count, (G) endometrial thickness, (H) luminal epithelial height and (I) lumen area fraction. (J) Distribution of oestrous cycle stages (proestrus, oestrus, metestrus, dioestrus) in Nv, Nv TMX treated, *Tb* and *Tb* TMX treated animals. Data from 2 independent experiments with mean ± SEM shown. Statistical analysis was performed using Kruskal-Wallis with Dunn’s *post-hoc* multiple comparisons (C, D) or one-way ANOVA (F-I). The supporting data for Fig 6C, D, F–J can be found in S5 Data in S1 File.

Since tamoxifen treatment could rescue the uterine atrophy, we next asked whether the uterine immune profile was also altered in response to tamoxifen exposure. Tamoxifen administration resulted in an increased frequency of CD45^+^ cells in infected animals (Fig 7A) and altered the myeloid composition of the uterus. Within infected animals, tamoxifen treatment increased monocyte frequency while reducing macrophage frequency (Fig 7B). Despite these changes, monocytes from infected animals retained elevated Ym1 expression (S10B Fig), while macrophages similarly maintained a type 1-skewed phenotype (CD11c^+^CD206^-^), with increased pro-inflammatory macrophages in infected groups irrespective of tamoxifen treatment (S10C Fig). Tamoxifen treatment also altered the cDC compartment, with an increased proportion of cDC2s (XCR1^-^CD11b^+^) in both naive and infected tamoxifen treated animals (S10D Fig). In contrast, eosinophil frequency significantly reduced during infection regardless of tamoxifen treatment (Fig 7B). Despite these changes in the myeloid compartment, the characteristic Th1 skewing associated with *T. brucei* infection (Fig 3) was unaffected by tamoxifen treatment. Infected animals exhibited an increased frequency of T cells irrespective of tamoxifen treatment (Fig 7B). T cells from infected animals also exhibited increased Ki67 expression regardless of tamoxifen administration (Fig 7C). Profiling of the CD4^+^ T cells revealed substantial Th1 skewing, characterised by increased T-bet expression in infected animals (Fig 7D), and reduced expression of Th2 and Th17 associated transcription factors GATA3 and RORγt, respectively (S10E and S10F Fig). *Ex vivo* PMA/ionomycin stimulation of udLN from infected animals exhibited enhanced IFNγ production (Fig 7E) compared to naive controls irrespective of treatment group. Collectively, these data demonstrate that *T. brucei* infection drives profound remodelling of the FRT, broad transcriptional changes across the HPG axis and skews the local uterine immune landscape towards a pro-inflammatory state. These changes were accompanied by altered oestrous cyclicity, ovarian and uterine morphology and circulating reproductive hormones. While tamoxifen treatment rescued uterine and ovarian atrophy, the uterine immune environment remained polarised, suggesting that the structural and immune changes are driven by different factors.

**Fig 7:**
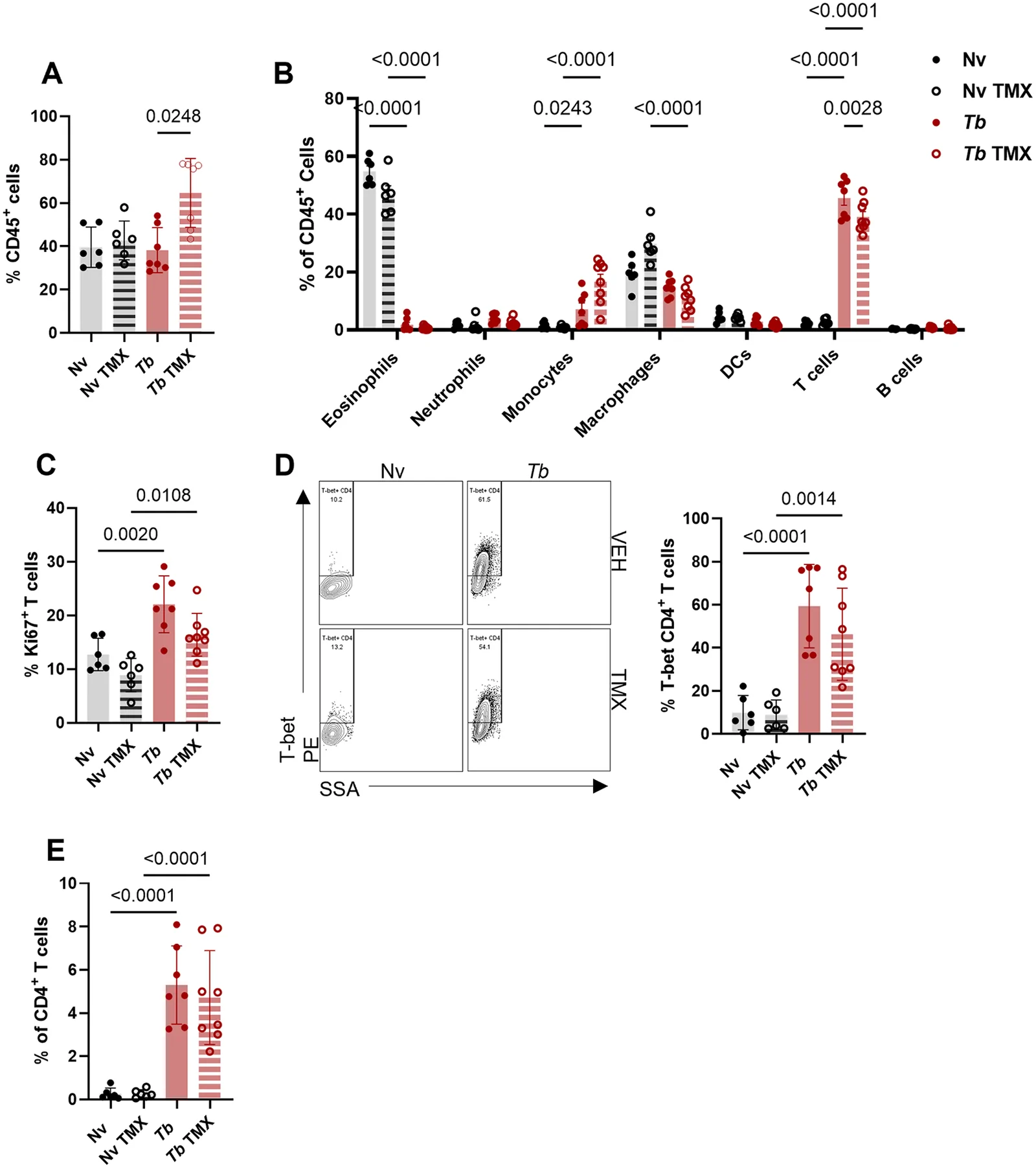
Tamoxifen treatment does not reverse uterine immune alterations during *T. brucei* infection. Flow cytometry analysis of uterine immune cell populations from naive, naive TMX-treated, *Tb* and TMX-treated *Tb* animals. (A) Proportion of uterine CD45^+^ cells. (B) Proportions of major myeloid cell populations as a proportion of total CD45^+^ cells. (C) Quantification of Ki67 expression in T cells across conditions as a percentage of total T cells. (D) Representative flow plots and quantification of T-bet expression by CD4^+^ T cells as a percentage of CD4^+^ T cells. (E) IFNγ production by udLN CD4^+^ T cells following PMA/ionomycin stimulation. Data from 2 independent experiments with mean ± SEM shown. Statistical analysis was performed using Kruskal-Wallis with Dunn’s *post-hoc* multiple comparisons (A), one-way ANOVA (C-E), or two-way ANOVA with multiple comparisons (B). The supporting data for Fig 7A and B can be found in S6 Data in S1 File.

## Discussion

Systemic inflammatory infections, including those caused by the extracellular protozoan parasite *Trypanosoma brucei*, are known to exert profound effects across multiple host tissues, including the brain [6–8], skin [9,10], adipose tissue [11,12], lungs [47] and testis [14,15]. In both humans and animal models, chronic *T. brucei* infection is associated with impaired reproductive function [30,31], yet how the infection impacts the FRT itself is poorly understood. Here, we show that *T. brucei* parasites are readily detectable within the FRT during both murine acute and chronic stages of infection. Chronic infection was associated with dramatic uterine and ovarian atrophy, disruption of the oestrous cycle, and broad transcriptional dysregulation across the HPG axis. In parallel, the uterine immune environment became skewed towards a type 1 immune landscape, characterised by an enrichment of IFNγ-producing CD4^+^ T cells as well as type 1-associated myeloid skewing during both acute and chronic infection. Finally, using pharmacological oestrogen receptor modulation, we demonstrate that while infection-induced uterine atrophy, and oestrous cycle disruption, are hormone-dependent, the altered uterine immune landscape persists independently of hormonal administration.

The detection of *T. b. brucei* parasites within the uterus throughout infection extends previous observations that *T. brucei* can invade and establish across a range of host tissues. The parasites were located within the endometrium, the specialised uterine mucosal layer responsible for blastocyst implantation and forming the maternal component of the placenta [57]. As animals in the initial experiments were not perfused prior to tissue collection, parasites within the uterine vasculature may have contributed to the *Pfr2* signal detected by qPCR. However, the persistence of parasite DNA following perfusion, together with the histological localisation of BiP-positive parasites within the endometrial stroma, supports parasite localisation within the uterine tissue itself. This raises the possibility that *T. brucei* infection could have direct consequences for implantation, pregnancy maintenance and vertical transmission. Earlier work using bioluminescent imaging demonstrated FRT colonisation during *T. brucei gambiense* infection and reported that dams give rise to 60–80% infected offspring [16]. Although congenital transmission of African trypanosomiasis has been reported in both experimental models and humans, fewer than 20 cases have been documented, potentially reflecting underdiagnosis and the association of infection with miscarriage and foetal loss [16,58–60]. Whether *T. brucei* infection similarly enables vertical transmission following uterine colonisation remains unknown and represents an important area for future investigation.

Tight control of the uterine immune environment is essential in balancing pathogen defence with reproductive maintenance [61,62]. Here, *T. brucei* infection induced substantial remodelling of the local immune landscape characterised by an enrichment of activated IFNγ-producing T cells and inflammatory myeloid populations. These findings were consistent with the type 1 associated immune skewing previously described in the skin, adipose tissue and central nervous system (CNS) during infection [8,10]. Additionally, recent work has demonstrated that *T. brucei-*associated fat wasting is dependent on CD4^+^ and CD8^+^ T cells at distinct stages, highlighting that T cell accumulation can contribute to tissue pathology [25]. Eosinophils were almost completely depleted from the uterus throughout infection, mirroring observations in other infected tissues and suggesting broader disruption of type 2-associated immune populations during *T. brucei* infection [47]. Uterine eosinophils are abundant during homeostasis and are thought to play important roles in tissue remodelling and protection against pathogens [63,64]. Therefore, this reduction may reflect impaired recruitment, reduced local survival or immune displacement by other immune cell populations. However, the mechanisms underlying eosinophil loss during *T. brucei* infection remain to be determined. A further intriguing finding was that infiltrating monocytes expressed Ym1 during acute and chronic infection. Although Ym1 has historically been associated with alternatively activated myeloid cells, it is increasingly recognised as an active immunoregulatory protein [65]. Given that parasites were detected in the uterus during both infection stages, Ym1 expression may therefore reflect local tissue remodelling in response to local parasite colonisation.

Dysregulation of the uterine and ovarian immune landscape can have serious implications for reproductive health. Successful implantation and pregnancy maintenance requires tightly regulated temporal changes to the uterine and ovarian immune landscape [66]. Although IFNγ plays an essential role in early pregnancy by assisting in uterine spiral artery remodelling, angiogenesis and maintenance of the decidual component [67,68], an excessive type 1 immune environment has been associated with preeclampsia, recurrent miscarriage, preterm labour and foetal growth restriction [69–74]. Consistent with this, administration of IFNγ during early pregnancy impaired implantation [75], while IFNγ-deficient females displayed reduced foetal pathology in models of *Toxoplasma gondii* and malaria infection [76,77]. Whilst relatively little is known regarding the direct effects of sustained IFNγ exposure within the uterus itself, IFNγ is recognised as a potent regulator of tissue inflammation, vascular barrier integrity and apoptosis across multiple mucosal tissues [78–80], Interestingly, IFNγ and sex steroid hormones exhibit bidirectional regulation, with excessive IFNγ capable of suppressing ovarian progesterone synthesis, whilst progesterone itself can negatively regulate IFNγ production [81,82]. This is in line with our findings of elevated uterine type 1 inflammation alongside reduced progesterone during chronic infection. Although reproductive complications during *T. brucei* infection are likely multifactorial and have largely been attributed to systemic inflammatory disease, damage of the CNS and endocrine disruption [40], our findings suggest that direct inflammatory remodelling of the uterine microenvironment may also contribute to impaired reproductive outcomes. This study provides the first transcriptional characterisation of the HPG axis across acute and chronic *T. brucei* infection. The HPG axis is implicated in trypanosome-associated hypogonadism [40,83], largely due to the established ability of *T. brucei* to invade and accumulate within the CNS [6,84]. Indeed, *T. brucei* parasites have been shown to occupy niches within the hypothalamus and anterior pituitary gland [85] and infection has been linked to significant histopathological damage at these sites [86]. More recently, single-cell and spatial transcriptomic analyses have demonstrated that *T. brucei* accumulates within and around hypothalamic-associated circumventricular organs, where parasite presence was associated with pronounced neuroinflammatory responses [87]. Extending these observations, we identified transcriptional dysregulation across the hypothalamus and pituitary gland that became progressively more pronounced as the infection advances. In the hypothalamus, dysregulated pathways were associated with endocrine regulation, metabolism, and immune responses. Given the profound adipose wasting observed during chronic infection and reported elsewhere [11], these findings suggest that chronic infection alters hypothalamic pathways involved in regulating energetic balance, which may contribute to reproductive dysfunction. The pituitary gland showed enrichment of immune activation pathways alongside alterations in neuronal and synaptic pathways. Together, these findings are consistent with the established neuroinflammatory response during chronic *T. brucei* infection and suggest that infection disrupts signalling across all components of the HPG axis [6]. Whether these changes reflect the damage caused by parasite invasion or as a response to systemic inflammation, a characteristic feature of experimental infection and of HAT, remains unclear and is likely a combination of both. However, pro-inflammatory cytokines are well-known suppressors of GnRH neuronal activity and gonadotropin release [88,89], where many receptors are shared between the HPG axis and the immune system [90].

Ovarian function is tightly regulated through signalling across the HPG axis, where pulsatile release of GnRH from the hypothalamus drives pituitary secretion of LH and FSH to support folliculogenesis, ovulation and steroidogenesis [36]. Despite the marked ovarian pathology, circulating LH and FSH concentrations were not significantly altered during chronic infection. However, as LH and FSH secretion occurs in pulsatile waves, endpoint hormone measurements may not fully capture infection-induced changes to pituitary hormone secretion. Nevertheless, ovarian responsiveness to gonadotropin signalling may be impaired locally, as expression of the LH receptor, *Lhcgr,* was significantly reduced. These findings suggest that impaired ovarian sensitivity to gonadotropin signalling may contribute to reproductive dysfunction during chronic infection. While transcriptional remodelling was evident throughout the axis, these changes differed between tissues, with endocrine, inflammatory, and metabolic pathways predominating in the hypothalamus, immune and neuronal pathways altered in the pituitary gland, and reproductive pathways most affected in the ovary. Reduced expression of *Lhcgr*, *Cyp11a1*, *Cyp17a1*, *Star* and *Hsd17b7* indicates suppression of ovarian steroidogenesis [49,50,55,91]. These transcriptional changes align with the observed reduction in corpora lutea numbers, increased follicular atresia, disrupted oestrous cyclicity, and reduced progesterone production, supporting the idea that chronic *T. brucei* infection drives ovarian dysfunction. Reproductive pathology has also been extensively reported during animal trypanosomiasis [92], particularly following *T. vivax* infection. Experimental infection has been associated with disrupted oestrous cycling and anoestrus, impaired ovarian function and reduced circulating progesterone across several livestock species [93–96]. More specifically, *T. vivax-*infected goats displayed reduced ovarian size, absence of corpora lutea and abnormal follicular development [97]. These changes closely resemble several of the reproductive abnormalities observed here during chronic *T. b. brucei* infection. Similar reproductive suppression has been described during other chronic infection settings, including *Toxoplasma gondii* [98,99] and *T. cruzi* infection [100,101], although the underlying mechanisms and direct effects on ovarian architecture are poorly characterised. Progression from proestrus to oestrus, and subsequent ovulation, is dependent on LH signalling through *Lhcgr* [91]. The predominance of chronically infected mice in proestrus stage, along with reduced *Lhcgr* expression suggests impaired ovulation (Fig 4A, 4B and 5F), which is reflected in the reduction in corpora lutea formation and progesterone production. Consistent with this, inactivating mutations in *Lhcgr* are associated with impaired fertility and ovulatory dysfunction in humans [102,103]. These changes may also contribute to the uterine atrophy observed during chronic infection. However, the persistence of uterine atrophy despite elevated circulating oestradiol suggests that altered ovarian hormone production is unlikely to be the sole driver of this phenotype.

Our finding that administration of tamoxifen recovered uterine atrophy and prevented cycle arrest at proestrus suggests that chronically infected mice retain the ability to respond to pharmacological modulation of oestrogen signalling. However, the uterine immune profile remained skewed towards a pro-inflammatory phenotype despite tamoxifen treatment, indicating that infection-driven uterine atrophy and immune remodelling are potentially separable processes. This suggests that the uterine immune response is not simply a downstream consequence of endocrine dysfunction. Instead, the factors driving uterine atrophy differ from those driving the uterine immune remodelling. Whether this is due to persistent systemic inflammation and/or local parasite presence remains unclear. Importantly, tamoxifen is not exogenous oestradiol, it is a SERM with agonistic activity in the uterus [56]. The ability of tamoxifen to restore uterine and ovarian size despite elevated endogenous oestradiol levels demonstrates that pharmacological modulation of oestrogen signalling can rescue infection-associated reproductive changes. Transcriptional analysis revealed unchanged *Esr1* expression in the ovary, although oestrogen receptor expression was not assessed in the uterus. However, *Pgr* expression was downregulated during both acute and chronic infection. Given the established role of progesterone signalling in ovulation and corpus luteum formation, this may further contribute to the impaired ovarian phenotype observed during chronic infection. Tamoxifen has been shown to increase parasitaemia in other models of chronic parasitic infection [104], but we did not observe this effect in *T. brucei*-infected mice. A limitation of the current study is that tamoxifen acts through selective modulation of oestrogen receptors and is known to cause uterotrophic effects [105]. Therefore, it is unclear whether restoration of uterine morphology reflects restoration of the infection-induced endocrine dysfunction or direct mitogenic activity of tamoxifen in the uterus. Similarly, although tamoxifen administration prevented accumulation of infected mice in proestrus, it remains unclear whether this reflects restoration of HPG axis function, or is due to the pharmacological effects of selective oestrogen receptor modulation [106].

An additional limitation is that circulating oestradiol concentrations were measured by ELISA, given the low circulating concentrations of oestradiol in mice, accurate quantification by ELISA is challenging due to limited sensitivity [107,108]. Whilst elevated oestradiol levels were observed during chronic infection, more sensitive analytical approaches such as LC-MS/MS will be required to confirm these findings [109]. Future studies using direct oestradiol and progesterone replacement approaches, along with longitudinal assessment of oestrous cycling and endocrine function, will therefore be important to further investigate the contributions of endocrine disruption and inflammation to FRT pathology during chronic *T. brucei* infection.

In addition to endocrine and immune disruption, infection-associated metabolic wasting may represent an important contributor to reproductive suppression. We and others have shown that chronically infected mice have altered systemic metabolic status [11,25], with profound adipose wasting. In support of this, we show that while body weight is not altered throughout infection, the body fat percentage of female mice is gradually lost throughout infection. Although weight loss is commonly reported during experimental *T. brucei* infection, much of the literature used male mice. Recent work directly comparing female and male mice demonstrated that this response is sexually dimorphic, with males exhibiting weight loss while females maintained body weight despite losing adipose tissue [11]. This highlights an important limitation of using body weight alone as a proxy for disease-associated wasting. Uterine atrophy developed alongside this loss of adipose tissue, suggesting that declining energy reserves may contribute to the uterine phenotype. Similar reductions in uterine size have been reported in rodent models of calorie restriction and during *T. gondii* infection, supporting the idea that adipose wasting contributes to reproductive suppression [98,110–112]. From an evolutionary viewpoint, suppression of reproductive function during chronic infection reflects a trade-off between energetically costly immune defence and reproduction [90]. Similar relationships between low energy availability, hypothalamic dysfunction and reproductive suppression have been established in non-infectious settings, including models of anorexia [28,113] and in cancer cachexia [114,115].

Our findings identify the female reproductive system as a major target of remodelling during *T. brucei* infection. We demonstrate parasite localisation within the FRT, profound uterine and ovarian pathology, HPG-axis transcriptional disruption and uterine immune dysregulation despite hormonal rescue. Together, this work provides direction for understanding how chronic trypanosome infection disrupts reproductive function through a combination of immune, endocrine, and metabolic mechanisms. Despite increasing recognition that infectious diseases can exert sex-specific physiological consequences [116], female reproductive health remains understudied across many NTDs. Therefore, these findings highlight the need to incorporate female reproductive health into studies of systemic infection and NTDs.

## Materials and methods

### Mice

Experiments were performed in female C57BL/6 mice obtained from either an in-house breeding colony or purchased from Envigo. Mice were randomly allocated into naive or infected groups by technicians independent of the research group. Experimental mice were maintained under specific pathogen-free conditions at the University of Manchester at 20–26℃ on a 12:12 hr light-dark cycle, with standard chow *ad libitum*, and were standardly used at 8–12 weeks. Experiments were approved under a project licence granted by the Home Office UK to Juan F. Quintana (PP5602024), reviewed by the University of Manchester, and performed in accordance with the United Kingdom Animals (Scientific Procedures) Act of 1986.

### Oestrous staging

A vaginal swab was collected from each animal with a cotton bud (TAMIYA) wetted with saline and inserted into the vagina. The swab was gently turned and rolled against the vaginal wall, removed and the cells were transferred onto a glass slide. Vaginal swabs were collected weekly from 09:00–11:00 to reduce variability. At the time of tissue harvest, vaginal lavages were collected by inserting 50 µl of PBS into the vagina, smeared onto Epredia SuperFrost Plus Adhesion slides, and allowed to air-dry before being fixed in ice-cold methanol and subjected to H&E staining as previously described [117]. The oestrous cycle stage of the stained sections was determined based on the proportions of cell types observed [118] (S7A Fig).

### *T. brucei* infection

Bloodstream forms of *T. brucei brucei* Antat 1.1E were expanded in an NSG (NOD.Cg-Prkdc^scid^Il2rg^tm1Wjl^/SzJ) donor mouse before infection of experimental animals [119]. NSG mice were inoculated intraperitoneally with ~2 × 10^3^ *T. b. brucei* Antat 1.1E parasites diluted in 1.5% glucose-PBS (gPBS) in a total volume of 200 µl. Parasitaemia was monitored 3 times a week by tail venesection, with blood examined by microscopy and the rapid “matching” method [120]. At peak parasitaemia (typically 5–7 days post-infection), infected blood was collected by cardiac puncture from donor mice into EDTA-coated tubes and diluted in sterile 1.5% gPBS. Parasite concentration was determined using a haemocytometer and adjusted to the desired inoculum. Experimental C57BL/6 female mice were infected intraperitoneally with ~2 × 10^3^ parasites in 200 µl gPBS. Following infection, parasitaemia was monitored at regular intervals by microscopic examination of tail vein blood smears. Mice were monitored throughout experiments using approved health score sheets and were humanely culled if wellbeing deteriorated or predefined humane endpoints were reached. Animals were culled by rising CO_2_ or cardiac puncture under terminal anaesthesia at day 7, to represent the acute stage of infection, and days 21–24 to represent the chronic stage of infection [13,41]. For the additional perfusion experiment, chronically infected animals were anaesthetised with isoflurane and transcardially perfused with 25–30 ml of ice-cold 1X PBS containing 0.025% EDTA. For tamoxifen (TMX) administration studies, animals were administered TMX via voluntary consumption to minimise stress associated with oral gavage [121], as previously described [122]. Briefly, mice were trained for 3 days using sweetened condensed milk alone. From days 19–21 post-*T. brucei* infection, infected C57BL/6 mice and naive controls were administered 100 µl of TMX daily, dissolved in corn oil (2 mg/mouse) and delivered in a 1:1:1 mixture of corn oil, water and sweetened condensed milk through a 1 ml syringe.

### DNA purification

Uterine tissues were harvested from non-perfused animals, and for the additional chronic infection experiment, perfused animals, and snap frozen. For parasite quantification, genomic DNA (gDNA) was isolated from 10-20 mg of uterine tissue using the DNeasy Blood and Tissue Kit (Qiagen) according to the manufacturer’s instructions. gDNA was eluted in 25 µl of EB buffer (Qiagen), and DNA concentration was measured using a NanoDrop ND-1000 spectrophotometer (Thermo Fisher Scientific). DNA samples were diluted to 4 µg/ml prior to downstream analysis.

### Uterine parasite burden quantification

To quantify *T. brucei* burden in the uterus, Taqman real-time PCR targeting the trypanosome *Pfr2* gene was performed. Reactions were carried out in a 25 μl reaction mix comprising 1X Taqman Brilliant III master mix (Agilent, Stockport, UK), 0.05 pmol/μl forward primer (CCAACCGTGTGTTTCCTCCT), 0.05 pmol/μl reverse primer (CGGCAGTAGTTTGACACCTTTTC), 0.1 pmol/μl probe (FAM- CTTGTCTTCTCCTTTTTTGTCTCTTTCCCCCT-TAMRA) (Eurofins Genomics, Germany) and 20 ng template DNA. A standard curve was constructed using a serial 10-fold dilution range: 1  ×  10^10^ to 1  ×  10^2^ copies of pCR2.1 vector containing the cloned *Pfr2* target sequence (Eurofins Genomics, Germany). The amplification was performed on an ARIAMx system (Agilent, USA) with a thermal profile of 95℃ for 10 min followed by 45 cycles of 95℃ for 15 s and 60℃ for 1 min and 72℃ for 1 s. The *T. brucei* load in the uterine samples was quantified by interpolation from the standard curve and expressed as an estimate of parasite burden.

### Body composition measurement

Total body lean and fat mass were measured before infection, and then weekly thereafter in naive and infected animals. The total fat mass was normalised to the weight of the individual mouse before infection. The parametrial adipose tissue associated with the FRT was dissected away from the uterus and weighed. The parametrial adipose tissue was normalised to body weight at the time of harvest.

### Histology

For histological analysis, 2 mm uterine sections were fixed in 10% neutral buffered formalin (NBF; Sigma-Aldrich) overnight, while ovaries were fixed overnight in Bouin’s solution (Sigma-Aldrich) before being transferred to 70% ethanol for storage. Tissues were subsequently dehydrated using a Leica ASP300 tissue processor (Leica Biosystems) prior to paraffin embedding. 5 μm sections of ovarian tissue were prepared using a Leica RM2255 rotary microtome and mounted onto Epredia SuperFrost Plus Adhesion slides. Sections were then scanned on a Pannoramic 250 slide scanner (3D Histech). Quantification was performed on a single ovarian section containing the largest cross-sectional area of each ovary and analysis was performed blinded to experimental group. For ovarian follicle counts, ovary sections were stained with Haematoxylin and Eosin (H&E). Follicles were classified according to morphological criteria as primordial/primary, secondary, antral and atretic follicles. Primordial and primary follicles were quantified as a single category. Corpora lutea were identified based on characteristic morphology [123,124]. Uterine sections were sectioned and stained by the Histology Research Service at the University of Glasgow veterinary school. Briefly, 3 µm uterine sections were prepared using a microtome (HM340E semi-automated, Epredia) and mounted onto Slidemate Plus Adhesion Microscope Slides (Epredia). For morphology analysis, uterine sections were stained with H&E. For BiP staining, sections were processed on a Leica BOND RX automated staining platform. Following deparaffinisation and heat-induced epitope retrieval (ER1, 20 min, 100℃), sections were incubated with anti-BiP antibody (1:1200, 30 min) followed by HRP-conjugated anti-rabbit secondary antibody and visualised using DAB. For CD3 staining, antigen retrieval was performed in sodium citrate buffer (pH 6) for 90 s at 124℃, followed by incubation with anti-CD3 antibody (DAKO A0452, 1:100, 30 min) and HRP-conjugated anti-rabbit secondary antibody. Staining was visualised using DAB and sections were counterstained with haematoxylin. Sections were scanned using a Zeiss Axioscan 7 microscope. Analysis of histology images was achieved using Qupath (v. 0.3.2) or Zen Lite (3.13).

### Murine cell isolation

Following culling by rising CO_2_ or cardiac puncture under terminal anaesthesia, the uterus was dissected from the peritoneal cavity and separated from the ovaries, cervix, and surrounding fat, and placed in complete media (RPMI 1640 (Sigma-Aldrich), 2 mM L-Glutamine, 100 µg/ml penicillin, 100 µg/ml streptomycin (Sigma-Aldrich), 1x NEAA (non-essential amino acids), 1 mM sodium bicarbonate (NaHCO3) (Sigma-Aldrich), 20 mM HEPES (Sigma-Aldrich), and 10% foetal calf serum (FCS)). Uterine index was represented as uterine weight as a proportion of body weight. Before tissue collection, mice were intravenously administered fluorescently labelled anti-CD45 (clone: 30-F11) to distinguish between blood circulating (i.v. CD45-BV785^+^) and uterine tissue cells (CD45-BV785^-^). Single-cell suspensions were obtained through mechanical and enzymatic digestion. Briefly, uterine tissues were finely minced and incubated in complete RPMI media containing collagenase D (0.625 mg/ml; Roche), collagenase V (0.425 mg/ml; Sigma-Aldrich), dispase II (1 mg/ml; Gibco), and DNase I type IV (0.03 mg/ml; Sigma-Aldrich) for 40 min at 37℃ under continuous agitation (200 rpm). Following incubation, digested samples were placed on ice and 500 μl of ice cold FACS buffer (PBS containing 2% FCS and 2 mM EDTA (Sigma-Aldrich)) was added to stop the enzymatic reaction. Digested samples were passed through a 70 μm nylon mesh filter with the aid of a 2 ml syringe plunger and topped up with FACS buffer to achieve a single cell suspension. Cells were pelleted by centrifugation (500 × *g*, 5 min, 4℃), the supernatant was discarded and cell pellets were transferred to a 96-well V-bottom plate. Uterine-draining lymph nodes were crushed through a 70 μm filter, washed with FACS buffer, centrifuged (500 × *g*, 5 min, 4℃), and resuspended in LN (lymph node) media (HBSS (Sigma-Aldrich), supplemented with 100 µg/ml penicillin, 100 µg/ml streptomycin (Sigma-Aldrich), 3% FCS, 2 mM L-Glutamine and 0.01% β-mercaptoethanol).

### *Ex vivo* stimulation of uterine-draining LN

To determine cytokine secretion potential from uterine-draining LNs, lymphocytes were stimulated with 30 ng/ml PMA (Sigma-Aldrich), 0.5 μg/ml ionomycin (Sigma-Aldrich), and 0.5 μl/ml GolgiStop (BD) in LN media (3 hr, 37℃) in a 96-well U-bottom plate in a final volume of 200 μl. Samples were subsequently processed for flow cytometry.

### Flow cytometry

Single cell suspensions were washed with PBS and stained for viability with Zombie UV (1:2000; BioLegend) (10 min, RT). Suspensions were then surface stained with a combination of antibodies (S1 Table) in FACS buffer with FcR block (1:200; BioLegend) for 30 min. Following surface staining, cells were washed twice in FACS buffer. For intracellular staining, cells were permeabilised in 50 μl BD cytofix/cytoperm (BD) for 1 hr and thoroughly washed with 1x eBioscience permeabilisation buffer (Thermo Fisher Scientific). Intracellular antibodies (S1 Table), diluted in 1x eBioscience permeabilisation buffer, were added overnight. The next day, cells were washed twice in permeabilisation buffer and resuspended in FACS buffer. Samples were acquired on a BD FACSymphony, and data were analysed using FlowJo version 10 (Treestar). Immune cell frequencies were calculated as a proportion of live, singlet cells expressing CD45, with individual immune populations expressed as a proportion of CD45^+^ cells. CD45^+^ cell numbers were calculated by multiplying the total viable cell count, determined by haemocytometer, by the percentage of CD45^+^ cells identified by flow cytometry, and were normalised to wet uterine tissue weight (cells/mg tissue).

### Hormone quantification by ELISA

Blood was collected from mice by cardiac puncture under isoflurane anaesthesia into EDTA-containing syringes and separated by centrifugation at 1000 × *g* for 10 min. Plasma was stored at −80℃ until analysis. Plasma LH (FineTest; EM1188), FSH (FineTest; EM1035) and oestradiol (R&D Systems Inc.; KGE014) concentrations were quantified using commercially available competitive ELISA kits according to the manufacturer’s instructions. All samples were analysed in duplicate, with standard curves and blank controls included. Absorbance was measured at 450 nm on an Infinite M200 PRO multimode microplate reader (Tecan Group Ltd.), with hormone concentrations interpolated from standard curves.

### Mass spectrometry

To each 50 µl plasma sample provided, gestodene D_6_ internal standard was added at a final concentration of 10 ng/ml. Steroids were extracted from the samples using a liquid-liquid extraction method based on previously described methods [125]. Briefly, 200 µl methyl *tert*-butyl ether and hexane at a ratio of 25 + 75, v + v, was added to each sample and mixed by vortexing followed by sonication in a water bath for 5 min. The steroid-containing organic phase was separated from the aqueous phase by centrifugation at 4000 × *g* for 3 min. The samples were incubated on dry ice for 5 min to freeze the aqueous phase, facilitating transfer of the organic phase to a new glass autosampler vial with 300 µl insert. Samples were taken to dryness using a speedvac centrifuge. To improve the sensitivity of the steroids in the mass spectrometer, they were derivatised to quaternary amines by resuspension in 50 µl 1M Girard’s Reagent T and incubated for 1 hr at room temperature [126]. To confirm retention times and transitions of target analytes, a mixture of progesterone, estrone, corticosterone and cortisol standards was run at the start of the batch. LC-MS analysis was performed using a SCIEX Exion LC system consisting of two AD high pressure gradient pumps, vacuum degasser, AC column oven and AC Autosampler, coupled to a SCIEX 7600 ZenoTOF Q-TOF mass spectrometer with TurboV Optiflow ion source running a 50 µm ESI probe. The system was controlled by SCIEX OS v3.0. A sample volume of 10 μl was injected onto a 50 μl sample loop. Injection cycle time was 1 min per sample. Separations were performed using a Phenomenex Kinetex EVO C18 column with dimensions of 150 mm length, 2.1 mm diameter and 1.7 μm particle size. Mobile phase A was water with 0.1% formic acid, and mobile phase B was 98 + 2 acetonitrile + water, with 0.1% formic acid. Separation was performed by gradient chromatography at a flow rate of 0.1 ml/min, starting at 5% B for 1 min, then ramping to 100% B over 6 min, hold at 100% B for 2 min, then back to 5% B. Re-equilibration time was 5 min. Total run time including 1 min injection cycle was 15 min. Solvent flow was diverted away from the mass spectrometer to waste for the first 2 min. The mass spectrometer was ran in positive mode under the following source conditions: curtain gas pressure, 50 psi; ionspray voltage, 5500 V; temperature, 400℃; ESI nebuliser gas pressure, 50 psi; heater gas pressure, 70 psi; declustering potential, 80 V. Data was acquired using the high resolution-multiple reaction monitoring (MRM-HR) transitions (S2 Table). Acquired data were checked in PeakView 2.2 and quantified using MultiQuant 3.0.3.

### RNA extraction and sequencing for bulk RNA sequencing

To isolate RNA, hypothalamus, pituitary gland and ovarian tissue samples were harvested and stored in RNAlater (Invitrogen) at −80℃. The samples were then homogenised in lysis tubes containing 0.5 ml Trizol using a Bead Mill homogeniser (Fisherbrand), followed by an acid-phenol chloroform extraction. RNA was precipitated using isopropanol, followed by two subsequent washes in 75% ethanol. 25 μl of RNase-free water was added to the pellet, and the solution was incubated at 55℃ for 15 min. The concentration of the resultant RNA was measured using a Nanodrop ND100 Spectrophotometer (Thermo Fisher Scientific). RNA integrity was assessed using an RNA Nano 6000 Assay Kit (Agilent Technologies) with a Bioanalyzer 2100 (Agilent Technologies). Samples with an RNA integrity number (RIN) of > 6.0 were qualified for RNA sequencing.

### Transcriptomics analysis

Total RNA was submitted to Novogene (S3 Table). Quality and integrity of the RNA samples were assessed using a 4200 TapeStation (Agilent Technologies) and then libraries generated using the Illumina Stranded mRNA Prep. Ligation kit (Illumina, Inc.) according to the manufacturer’s protocol. Briefly, total RNA (typically 0.025-1 µg) was used as input material from which polyadenylated mRNA was purified using poly-T, oligo-attached, magnetic beads. Next, the mRNA was fragmented under elevated temperature and then reverse transcribed into first strand cDNA using random hexamer primers and in the presence of Actinomycin D (thus improving strand specificity whilst mitigating spurious DNA-dependent synthesis). Following removal of the template RNA, second strand cDNA was then synthesised to yield blunt-ended, double-stranded cDNA fragments. Strand specificity was maintained by the incorporation of deoxyuridine triphosphate (dUTP) in place of dTTP to quench the second strand during subsequent amplification. Following a single adenine (A) base addition, adapters with a corresponding, complementary thymine (T) overhang were ligated to the cDNA fragments. Pre-index anchors were then ligated to the ends of the double-stranded cDNA fragments to prepare them for dual indexing. A subsequent PCR amplification step was then used to add the index adapter sequences to create the final cDNA library. The adapter indices enabled the multiplexing of the libraries, which were pooled prior to loading on to the appropriate flow-cell. This was then paired-end sequenced (59 + 59 cycles, plus indices) on an Illumina NovaSeq6000 instrument. Finally, the output data was demultiplexed and BCL-to-Fastq conversion performed using Illumina’s bcl2fastq software, version 2.20.0.4 (S4 Table).

Read counts were filtered to remove low abundance transcripts. Hypothalamus, pituitary and ovary datasets were analysed independently throughout, with transcripts with > 250 total reads within each dataset were retained (S5 Table). Read count normalization and differential expression analysis were performed using ‘DESeq2’ (v1.50.2). Log fold change shrinkage was performed using the ‘ashr’ package (v2.2-63). Transcripts were considered significantly differentially expressed where the adjusted *p* value was < 0.05 and the absolute log2 fold change was > 1 (S6 Table). Principal components analysis (PCA) was performed on the 250 most variable transcripts using the ‘prcomp’ function, following variance stabilizing transformation of the normalized read counts. For gene cluster analysis, significantly differentially expressed transcripts present in at least one statistical comparison were selected. Transformed read counts were then scaled across samples using the ‘scale’ function. Hierarchical clustering of transcripts was then performed using the ‘hclust’ and ‘dist’ functions. Gene clusters were then generated using K-means clustering where k = 5. The value for k was guided by within groups sum of squares analysis. A heat map of the scaled values was generated and annotated with the top 5 differentially expressed transcripts per cluster based on lowest adjusted p value. Line graphs showing the per-cluster gene expression pattern were generated by calculating the within-cluster median scaled value for each group. Gene set enrichment analysis of Gene Ontology (GO) Biological Process (BP) terms was then performed using the ‘compareCluster’ function from the ‘clusterProfiler’ package (v4.18.4) with minimum gene set size set to 50. Redundant GO-BP terms were then filtered using the ‘simplify’ function with cutoff set to 0.3. The top 5 enriched terms per cluster were selected by adjusted p value (S7 Table).

### Statistical analysis

Statistical analyses were performed using Prism 10 (GraphPad Software). Data were assessed for normality using the Shapiro-Wilk test. For comparisons between two groups, an unpaired t-test or Welch’s t-test was used when data were normally distributed. For non-normally distributed data, or when sample size was insufficient, a Mann-Whitney test was used. For comparisons involving three or more groups, a one-way or two-way ANOVA was performed when normality assumptions were met, followed by the appropriate multiple comparison test. For non-normally distributed data involving three or more groups, a Kruskal-Wallis test followed by Dunn’s multiple comparisons test was used. Differences in oestrous cycle stage distributions were analysed using Fisher's exact test. Exact *p* values are reported in the figures. Mean values ± SEM are shown. Raw data were combined from 2-5 individual experiments with n = 3–5 per group.

## Supporting information

S1 Fig Parasite DNA is detected in uterine tissue following perfusion during chronic *T. brucei* infection. (A) Representative image of 3,3’-diaminobenzidine (DAB) immunostaining against the *T*. *brucei*-specific protein BiP in uterine cross section from acute and chronically infected females (scale bar 20 µm;; brown signal). (B) *T. brucei* burden was estimated in uterine tissue following perfusion by qPCR detection of the parasite-specific *Pfr2* gene in naive and chronically infected females. Data are from one experiment with mean ± SEM shown. Statistical analysis was performed using a Mann-Whitney test (B).). BiP = Binding-Immunoglobulin protein. The supporting data for S1A and B Fig can be found in S7 Data in S1 File.
(TIF)

S2 Fig Myometrial thickness is unchanged by *T. brucei* infection. Quantification of uterine histological sections (Fig 2D) from naive, acutely- and chronically infected mice. (A) Outer myometrial width. (B) Inner myometrial width. Data from 2 independent experiments with mean ± SEM shown. Statistical analysis was performed using Kruskal-Wallis with Dunn’s *post-hoc* multiple comparisons (A, B). The supporting data for S2A–D Fig can be found in S8 Data in S1 File.
(TIF)

S3 Fig Body weight and composition changes in naive and *T. brucei*-infected mice. (A) Percentage change in body weight relative to pre-infection baseline in naive and *T. brucei* infected mice over time. (B) Lean mass as a percentage of total body weight measured by MRI. (C) Liver weights as a proportion of body weight. (D) Spleen weights as a proportion of body weight. Data combined from 2 independent experiments with mean ± SEM shown. Statistical analysis was performed using one-way ANOVA (C) or Kruskal-Wallis with Dunn’s post-hoc multiple comparisons (D).
(TIF)

S4 Fig Flow cytometric gating strategy to identify major immune cell subsets. Example gating strategy from adult female naive mice. Cells were isolated from whole uterine digests, stained and defined based on their expression profile of cell-surface and intracellular antigens/transcription factors. Following the exclusion of debris, doublets and CD45^-^ cells, the remaining cells were taken forward to isolate specific myeloid populations (naive mouse) and lymphocytes (*Tb* chronic mouse represented for clarity) showing intracellular transcription factor staining with associated fluorescent minus one (FMO) controls. The supporting data for S4A–G Fig can be found in S9 Data in S1 File.
(TIF)

S5 Fig Local uterine immune responses during *T. brucei* acute and chronic infection. Flow cytometry data of immune cell populations in the uterus of naive (black; Nv), acute *T. brucei* infection (light red; *Tb* acute) and chronic *T. brucei* infection (burgundy; *Tb* chronic). (A) Whole uteri CD45 counts per mg of tissue. (B) Percentage of NK cells as a proportion of CD45^+^ cells in naive and chronically infected animals. (C) Ym1 expression by monocytes. (D) Proportion of uterine CD206^-^CD11c^+^ macrophages as a proportion of total macrophages. (E) Proportion of cDC1s and cDC2s as a proportion of total cDCs. (F) Proportions of CD4^+^ and CD8^+^ T cells as a proportion of total CD45^+^ cells. (G) GATA3 expression by CD4^+^ T cells as a percentage of CD4^+^ T cells. (H) RORγt expression by CD4^+^ T cells as a percentage of CD4^+^ T cells. (I) IL-13 cytokine production by CD4^+^ uterine T cells following PMA/ionomycin stimulation. (J) Representative uterine cross sections of CD3^+^ DAB staining across Nv, *Tb* acute and *Tb* chronic conditions (scale bar = 50 µm). Data combined from 4-5 independent experiments with mean ± SEM shown. Statistical analysis was performed using one-way ANOVA (A, H), Mann-Whitney test (B), Kruskal-Wallis with Dunn’s *post-hoc* multiple comparisons (C, D, G, I) or two-way ANOVA with multiple comparisons (E, F).
(TIF)

S6 Fig *T. brucei* acute and chronic infection drives systemic Th1 polarisation. Flow cytometry data of T cell populations in the uterus of naive (black; Nv), acute *T. brucei* infection (light red; *Tb* acute) and chronic *T. brucei* infection (burgundy; *Tb* chronic). (A) Representative flow cytometry plots of T cell CD45 IV^+^ staining across conditions. (B) GATA3, T-bet and RORγt expression by CD4^+^ T cells as a percentage of CD4^+^ T cells. (C) Proportions of CD4^+^ and CD8^+^ T cells as a proportion of total CD45-BV785^+^ cells. Data combined from 2-3 independent experiments with mean ± SEM shown. Statistical analysis was performed using two-way ANOVA with multiple comparisons (B, C). The supporting data for S6B and S6C Fig can be found in S10 Data in S1 File.
(TIF)

S7 Fig Chronic *T. brucei* infection drives changes to reproductive cycling. (A) Mice were culled and vaginal smears were collected to identify the oestrous cycle stage (scale bar = 50 μm). Proestrus stage is predominantly nucleated epithelial cells (black arrows). Oestrus stage consists of enucleated cornified epithelial cells (grey arrows). Metestrus stage consists of nucleated and enucleated epithelial cells and leukocytes (white arrow). Dioestrus stage mainly consists of leukocytes. (B) Number of corpora lutea (CL) per ovary. (C) Levels of circulating luteinising hormone (LH) and (D) follicle-stimulating hormone (FSH) in naive and *T. brucei* chronically infected females. Data combined from 2-3 independent experiments with mean ± SEM shown. Statistical analysis was performed using Mann-Whitney test (B-D). The supporting data for S6B–D Fig can be found in S11 Data in S1 File.
(TIF)

S8 Fig Acute and chronic *T. brucei* infection drive transcriptional changes in the hypothalamus and pituitary gland. Heat map representing the relative expression of (A) Hypothalamus and (B) pituitary gland genes identified as statistically differentially expressed in at least one comparison. Genes were grouped into clusters by K-means clustering. A summary of the median scaled expression of all genes from each cluster across Nv, *Tb* acute- and *Tb* chronic infections. Gene set enrichment analysis of gene clusters using GO Biological Process terms.
(TIF)

S9 Fig Tamoxifen treatment during *T. brucei* infection does not alter infection. (A) Number of parasites per ml of blood from C57BL/6 female mice infected with ~2000 *T. b. brucei* parasites and treated with vehicle or tamoxifen (TMX) from day 19 onwards (dotted line). Parasitaemia was measured using phase microscopy and the rapid ‘matching’ method. Dotted line on y axis is limit of detection. (B) Representative image of immunohistochemistry staining the *T*. *brucei*-specific protein BiP in uterine cross section from untreated (left panel) and TMX (right panel) treated chronically infected females (scale bar = 20 µm). (C) Liver and (D) spleen weights as a proportion of body weight. (E) Parametrial fat weight as a proportion of body weight. Data from 2 independent experiments with mean ± SEM shown. Statistical analysis was performed by two-way ANOVA (A) or one-way ANOVA (C-E). The supporting data for S9A, B and D–F Fig can be found in S12 Data in S1 File.
(TIF)

S10 Fig Tamoxifen treatment during *T. brucei* infection does not recover infection-induced alterations to uterine immunology. (A) Total uterine CD45^+^ counts per mg of uterine tissue. (B) Ym1 expression by monocytes. (C) Proportion of uterine CD206^-^CD11c^+^ macrophages as a proportion of total macrophages. (D) Proportion of cDC1s and cDC2s as a proportion of total cDCs. (E) GATA3 expression by CD4^+^ T cells as a percentage of CD4^+^ T cells. (F) RORγt expression by CD4^+^ T cells as a percentage of CD4^+^ T cells. Data from 2 independent experiments with mean ± SEM shown. Statistical analysis was performed using one-way ANOVA with multiple comparisons (A-C, E, F) or by two-way ANOVA (D). The supporting data for S10A-E Fig can be found in S13 Data in S1 File.
(TIF)

S1 Table Antibodies used for flow cytometry. (XLSX)

S2 Table High resolution-multiple reaction monitoring (MRM) transitions used for steroid quantification by LC-MS/MS. (XLSX)

S3 Table BulkRNA sequencing metadata. Metadata for all samples included in transcriptomic analysis.
(XLSX)

S4 Table BulkRNA sequencing quality control summary. (XLSX)

S5 Table Raw RNA sequencing read counts. (XLSX)

S6 Table Differential gene expression analysis results. (XLSX)

S7 Table Gene ontogeny biological processes (GO-BP) enrichment analysis results. (XLSX)

S1 File S1 Data. T. brucei accumulates in the uterus. S2 Data. Parasite accumulation correlated with uterine and ovarian atrophy during chronic phase. S3 Data. At acute and chronic infection stages, T. brucei drives type 1 skewing of the uterine immune landscape. S4 Data. Chronic T. brucei infection drives hypogonadism. S5 Data. Hormone replacement therapy (HRT) recovers uterine atrophy. S6 Data. Hormone replacement therapy (HRT) does not alter uterine immunological profile during T. brucei infection. S7 Data. Myometrial thickness is unchanged by T. brucei infection. S8 Data. Longitudinal body weight and lean mass changes in naive and T. brucei-infected mice. S9 Data. Local uterine immune responses during T. brucei acute and chronic infection. S10 Data. T. brucei acute and chronic infection drives systemic Th1 polarisation. S11 Data. Chronic T. brucei infection drives changes to reproductive cycling. S12 Data. Hormone replacement therapy (HRT) during T. brucei infection does not alter infection. S13 Data. Hormone replacement therapy (HRT) during T. brucei infection does not recover infection-induced alterations to uterine immunology.
(XLSX)
