# Supplementary material for: Trypanosoma brucei infection remodels the uterine immune environment and drives neuroendocrine dysfunction
Source: PLoS Pathog. 2026 Oct 9;22(10):e1014667. doi: 10.1371/journal.ppat.1014667 (PMC13671655; doi:10.1371/journal.ppat.1014667)
Supplement: Supplementary file 1 [file ppat.1014667.s020.docx]

**Decision on PloS Pathogens: PPATHOGENS-D-26-01488**

We thank the Academic Editor and reviewers for their assessment, overall support of our work and helpful comments. Below, we have summarised the changes made to the manuscript. We believe the textual revisions, additional analyses and supplementary experiments have further strengthened the manuscript and have satisfactorily addressed the reviewers’ comments. We hope that this manuscript kick-starts further conversations around this exciting yet underexplored area of research and believe that it will be of interest to the broader PLoS Pathogen readership.

**Reviewer #1: Major concerns**

Reviewer #1: 1. Sup Fig 8B There is considerable variation in the Pfr2 copies/mg tissue in non-tamoxifen treated infected animals, which might be skewing the statistical analysis. I would recommend increasing the N of this experiment to confirm its findings.

We thank reviewer 1 for highlighting this point and agree the graph shows considerable variation. Due to the length and complexity of tamoxifen experiments, it was not possible to repeat the tamoxifen experiments within the timeframe of the revision period. We have therefore removed the *Pfr2* copies/mg tissue for the tamoxifen experiments and instead retained histology analysis, which allows direct visualisation of parasite localisation within the uterine tissue.

2. Lines 416-418: the authors acknowledge that the mice were not perfused prior to tissue collection. This is a red line when discussing tissue tropism. Based on previous literature reporting tissue load vs. parasitaemia, the lack of perfusion will considerably overestimate parasite burden. Based on this point and the previous one, I suggest that claims of tropism to the FRT to be toned down or supported with actual parasite quantification in the FRT extravascular spaces. Currently, only a representative H&E stain is provided. Considering this and the high variation in the Pfr2 RT-qPCR (Sup Fig 8B), it is my recommendation that an experiment is conducted to assess parasite burden in perfused FRT.

We thank reviewer 1 for this point. We have now included data from perfused chronically infected animals in supplementary Figure 1 (Sup. Fig. 1B) and added further representative histology sections to the supplementary from animals during acute and chronic infection (Sup. Fig. 1A). In the original non-perfused animals, uterine parasite DNA was significantly higher during chronic than acute infection. In the perfused animals, parasite DNA remained detectable in chronically infected uteri (see figures below; left is not perfused and right is perfused). However, we have softened the language throughout the manuscript to indicate parasite colonisation as opposed to tropism.

Non-perfused

Perfused

**Lines:** 93, 410 and 413.

3. Line 347: Why was tamoxifen administered by voluntary feeding rather than standard injection and how can the authors be sure the amount of tamoxifen ingested was similar between naïve and infected groups? I think this is particularly relevant given that T brucei infection is often associated with periods of anorexia where food intake is significantly reduced.

We thank reviewer 1 for this point and we apologise for the misunderstanding arising from our wording. In our experience, oral gavage in chronically infected mice tend to result in poor health outcomes and sometimes lethality. We have therefore opted to use a refined approach considering animal welfare. For this, the animals were administered tamoxifen orally in a palatable sweetened condensed milk solution to favour voluntary feeding. Animals were briefly restrained while the solution was administered and until the full dose had been consumed. The dose was therefore controlled and not given *ad libitum*. We have altered the wording to better reflect this. We have also added references supporting the use of this approach as a less stressful alternative to i.p. injection or oral gavage [1,2].

**Lines:** 642-648.

**Reviewer #2: Major concerns:**

The characterization of uterine immune cell infiltration is presented in an unclear and unconventional manner. The authors should present total counts and frequencies of CD45+ leukocytes, together with all subpopulations, within the same figure (Figures 3 and 7). To clearly demonstrate the claimed differences, total CD45+ leukocyte counts and the frequencies of myeloid and lymphoid subsets (eosinophils, neutrophils, monocytes, dendritic cells, B cells, and T cells) should be consolidated into a single figure. Moreover, it is puzzling that the authors do not report NK cell data, given their well-established importance in uterine physiology and reproduction. In which stage of the estrous cycle were these analyses performed? Besides, the combination of markers used for the identification of each leukocyte subset by flow cytometry is not clearly detailed in the manuscript.

We thank reviewer 2 for highlighting this. We have now updated both figures 3 and 7 to include: total leukocyte frequency (with counts in the supplementary) along with myeloid and lymphoid proportions. We have also included NK cells from naive and chronically infected animals, showing a reduction during infection. We have updated the text describing the immune changes.

The animals were analysed at their naturally occurring oestrous stage because infection profoundly altered cycling and therefore stage matching was impractical. Oestrous stage was recorded for each animal at harvest, allowing us to consider cycle stages when interpreting the data. If requested by reviewer 2, we can indicate the oestrous stage of individual animals using different symbols in the relevant graphs.

Although we considered oestrous synchronisation with medroxyprogesterone acetate, as this is anti-inflammatory itself, administration could alter the uterine immune profile and infection course [3]. The combination of markers used to identify each population is detailed in the results, additionally we have shown the gating scheme to identify each population (Sup. Fig. 4).

**Lines:** 202-230, 363-380.

The authors hypothesize that uterine and ovarian atrophy (which co-occurred with increased serum estradiol levels alongside significantly reduced progesterone levels) resulted from impaired uterine responsiveness to endogenous estrogens. Consequently, they tested the efficacy of tamoxifen, an estrogen receptor agonist in the uterus and ovaries, in reversing the observed changes. Although interesting, this hypothesis is rather peculiar given that systemic estrogen levels were already elevated while progesterone levels were significantly reduced. Furthermore, transcriptional analyses revealed unchanged Esr1 expression in the ovary. Would it not have been more rational to hypothesize defects resulting from progesterone deficiency and, accordingly, test progesterone replacement therapy? In that regard, authors sustain that tamoxifen treatment reverted uterine pathology and estrous cycling. Were those analyses performed in females staged for estrous cycle phase?

We thank reviewer 2 for raising this point. We agree that the original wording may have implied that the phenotype resulted from oestrogen deficiency, which was not directly demonstrated. Tamoxifen was not used to correct for low circulating oestradiol concentrations. Instead, it was used as a pharmacological tool to help determine whether activation of oestrogen-receptor signalling was sufficient to restore reproductive phenotypes during chronic infection. We have revised the manuscript throughout to clarify this distinction and no longer describe the findings as evidence of impaired responsiveness to endogenous oestrogen or describe tamoxifen treatment as hormone replacement therapy.

**Lines:** 534-547, 132, 402.

We agree that unchanged *Esr1* expression in the ovary does not support reduced receptor expression as the mechanism underlying the phenotype. However, we did not quantify *Esr1* or *Esr2* expression within the uterus and therefore cannot determine whether altered uterine oestrogen receptor expression contributes to the phenotype. We have removed wording suggesting impaired responsiveness to endogenous oestrogen and instead state that tamoxifen can rescue the uterine and ovarian atrophy during infection.

Additionally, we agree that the reduction in progesterone levels provides a clear reason for progesterone replacement studies. We have acknowledged this as a limitation in the discussion and highlight direct progesterone replacement as an important future experiment.

**Lines:** 567-570.

Animals were not synchronised, and tissues were analysed irrespective of cycle stage. Oestrous stage was recorded for each animal at harvest, allowing us to consider whether the observed changes were associated with cycle stage. If requested by reviewer 2, we can indicate the oestrous stage of individual animals using different symbols in the relevant graphs. Chronic infection itself disrupted cycling and caused an overrepresentation of proestrus, making stage matching impractical. Tamoxifen is also known to disrupt the normal oestrous cycle [4]. The outcomes of the oestrous stages are shown in Fig. 6F.

Although interesting, the presented data do not clearly demonstrate that the infection decreased reproductive potential. Mating experiments assessing potency, fecundity, and fertility index, as well as rates of pre- and post-implantation embryo loss, would significantly strengthen the quality of the manuscript.

We thank reviewer 2 for this comment, we agree that directly measuring reproductive capacity would strengthen this manuscript. However, these experiments were beyond the scope of the current work and are not permitted on our current animal licence. Furthermore, chronic *T. brucei* infection results in substantial systemic illness, and therefore, extending the infection to perform mating studies would have raised significant animal welfare concerns. We have therefore moderated our conclusions throughout the manuscript to avoid inferring reduced fertility directly.

**Lines:** 381-388, 595-597.

**Reviewer #1: Minor concerns**

1. Lines 190-193: Despite the common splenomegaly and hepatomegaly, weight loss is usually reported during T. b. b. infection. Could the authors speculate on why that was not observed here? I think it is worth noting in the text this discrepancy with current literature. A few thoughts:

- Was there evidence for fluid accumulation in the lungs of infected mice that could also contribute to the unchanged body weight?
- Could female be more resistant to cachexia/muscle wasting than males (given that the majority of reports have used male mice)?

We thank reviewer 1 for this insightful comment. We have expanded the discussion to address the discrepancy with previous reports of weight loss during experimental *T. brucei* infection. Although overall body weight remained stable in our study, infected females exhibited marked reductions in body fat despite hepatosplenomegaly, indicating substantial alterations in body composition (Fig 2I and 2J). Although weight loss is commonly reported, much of the existing literature has used male mice. However, recent work by our lab, directly comparing male and female mice, has demonstrated that weight loss during chronic *T. brucei* infection is sexually dimorphic, with male mice exhibiting significant weight loss whereas females largely maintain body weight despite adipose tissue wasting [5]. We have now incorporated this discussion. We also note that we did not assess tissue oedema or fluid accumulation and therefore cannot exclude a contribution of altered fluid balance.

**Lines:** 574-581.

2. Lines 202-204: Can the authors explain what is the difference between the frequency of CD45+ cells (changed) and the total numbers of CD45+ cells / mg tissue (unchanged)? How is the frequency calculated and what could explain this discrepancy? Is it linked to the infection-induced uterine atrophy? If so, is there a true expansion/recruitment of leukocytes to the FRT if the absolute numbers of cells remain unchanged? And how does total number of cells remain unchanged if there is a clear expansion of CD3+ cells (described in subsequent sentences)?

We thank reviewer 1 for highlighting this point. CD45^+^ frequency was calculated as the proportion of live, singlet cells expressing CD45, whereas CD45^+^ cell numbers per mg of uterine tissue were calculated by multiplying the total viable cell count, determined by haemocytometer, by the percentage of CD45^+^ cells identified by flow cytometry, and normalising this to the wet weight of the uterine tissue. We have added this clarification to the Methods section.

**Lines:** 741-746.

This discrepancy could be due to a reduction in CD45^-^ cells in the atrophic uteri. We also agree that the increased frequency of CD45⁺ cells, despite unchanged CD45⁺ cells per mg, does not necessarily indicate an overall expansion of the uterine leukocyte compartment. Instead, this supports a relative enrichment of leukocytes within the recovered cell proportion alongside changes in the composition of individual immune cell populations, including the marked depletion of eosinophils. These data suggest remodelling of the uterine immune compartment, whereby eosinophils, which represent a large proportion of leukocytes in naive uteri, are reduced during infection, while T cells become more prominent (Figure 3B).

3. Lines 220-223: There is no significant increase in CD4+ T cells during acute infection. I suggest the sentence should be improved for clarity. Moreover, would it be possible to show the full composition of the immune repertoire rather than just CD4 and CD8+ T cells? Assuming the authors already have these data (alluded, for example, in the no. of macrophages and neutrophils), it would be helpful to have a weighted bar chart in naïve, acute and chronic with all CD45+ cells and the proportion of each cell subset assessed.

We thank reviewer 1 for this point and suggestion for presentation. We apologise that the original wording was unclear. Our analysis identified a significant increase in CD4^+^ T cells during acute infection however, we have revised the sentence to refer to the increase in total T cells as presented in Fig 3B. We have also revised Figures 3 and 7 to provide a broader overview of the major immune cell populations across naive, acute and chronic infection, including eosinophils, neutrophils, monocytes, macrophages, dendritic cells, T cells and B cells. CD4^+^ and CD8^+^ T cell frequencies are now shown separately in the corresponding supplementary figures, alongside additional immune cell phenotyping (Sup. Fig. 5I).

4. Still in the previous line, why were only chronically infected animal subjected to this treatment? Is there a reason to not assess acute infection? Given that all other figures so far have included both stages of infection, it would make sense to include it here too.

We thank reviewer 1 for this point. Since the FRT pathology and endocrine disruption were predominantly observed during chronic infection, we focused the tamoxifen studies on this stage where there was a clear pathological phenotype to rescue.

5. Fig6J and lines 362-365: the cycle profiles with tamoxifen do not reproduce a naïve animal. It would perhaps be more accurate to state that “during infection, and despite altered oestrous cycle, the HPG axis partially retains the capacity to respond to oestrogenic signals”.

We thank reviewer 1 for this point. We agree that the oestrous cycle profiles following tamoxifen treatment do not fully recapitulate those of naive animals and have revised the text accordingly.

**Lines:** 356-358.

6. Throughout the intro and results sections and very clearly on the concluding sentence of the results sections (lines 392-3), it appears that the authors would expect immune remodeling to be driven by hormonal disruption. Is this the case? If so, why would it be expected? If not, then I’d suggest rephrasing.

Immune and structural cells throughout the FRT express oestrogen and progesterone receptors and are therefore responsive to changes in circulating hormones. This is supported by the well-established changes in uterine immune cell populations across the oestrous cycle and by our observation that tamoxifen administration alters the uterine immune profile in naive animals. However, we believe that it is the combination of local parasite infiltration, systemic immune modulation, endocrine and metabolic changes that drive the uterine immune changes. Therefore, we have altered the language used to better reflect this.

**Lines:** 381-388.

7. Lines 411-413: I think it is overall agreed in the field that T. brucei can infect and establish in all organs. This has been reported in the early studies by Ikede, Losos and others in the 70s, but also recently by, for example, De Niz et al 2021. So, one could argue that the FRT does not come as an “unappreciated site of … tropism”, but it is rather an organ that, like all others observed to date, does not offer any effective barrier for parasite invasion. Whether we consider tropism as the ability of a microenvironment to support infection, or a preference of the microorganism for a given microenvironment is debatable, but I have seen no evidence in this paper to suggest that the FRT is a preferential site of parasite colonization.

We thank the reviewer for this insight and agree that the use of the word tropism perhaps overstates our findings. We have removed this terminology throughout the manuscript and revised the text to state that *T. brucei* can invade and establish within the FRT, consistent with its ability to colonise a broad range of host tissues.

**Lines:** 93, 408 and 414.

8. Lines 626-639: Following the point above, the title and description of this methods section should be corrected to acknowledge that what is measured is the total parasite burden in the uterus and associated vasculature, and not the tissue parasite burden.

We thank reviewer 1 for highlighting this. We agree that, for non-perfused animals, the qPCR analysis measures total parasite burden within the uterus and associated vasculature rather than tissue-specific parasite burden. We have therefore revised the methods and corresponding terminology throughout the manuscript to reflect this. We have also added details of the perfusion protocol used for the additional chronic infection experiment, in which parasite burden was quantified following perfusion to minimise the contribution of parasites within the uterine vasculature.

**Lines:** 637-656.

9. Lines 424-427: The authors mention that congenital transmission has been reported in both experimental models and humans, but cite papers 58, 59, and 60. Ref 58 and 60 are two human reports, ref 59 is a short review that does not (to my knowledge) include any reference to experimental models. Is there a reference missing? I think it would also be important to acknowledge how many cases have been reported, because it appears to be less than 20 cases reported in almost a century (1933?). So, whilst I don’t argue that it might be underreported, I also consider that, if it were relatively frequent, sleeping sickness with such a severe clinical presentation would be hard to miss.

We thank reviewer 1 for bringing this to our attention. The reviewer is correct that a reference to an experimental model of congenital transmission was missing. We had intended to cite Biteau et al., 2016, who demonstrated vertical transmission in an experimental *T. b. gambiense* infection model, and this reference has now been added. We have also revised the text to acknowledge the small number of congenital cases reported in humans. While congenital transmission appears to be rare, this may reflect underdiagnosis as well as the association of African trypanosomiasis with miscarriage and foetal loss, which may limit the number of pregnancies progressing to a stage at which congenital transmission can be observed.

**Lines:** 418-424.

10. Lines 427-429: Have the authors tried to breed infected mice to assess evidence for congenital transmission?

Thank you for this experimental idea. While we agree that such experiments would provide a direct assessment of fertility and congenital transmission, they were beyond the scope of the current work and are not permitted by our current animal licence. Furthermore, chronic *T. brucei* infection results in substantial systemic illness, and therefore, extending the infection to perform mating studies would have raised significant animal welfare concerns.

11. Still on the topic of congenital transmission and FRT remodeling, and especially given that the paper uses T. brucei brucei, an agent of nagana and not sleeping sickness, I am surprised papers describing T vivax-induced FRT changes are not cited nor discussed. I think discussing previous work on how T vivax affects the FRT (in a natural host) would strengthen the discussion by establishing precedent (ref: [https://doi.org/10.1016/j.vetpar.2012.08.001 [doi.org]](https://urldefense.com/v3/__https:/doi.org/10.1016/j.vetpar.2012.08.001__;!!PDiH4ENfjr2_Jw!G56s9wbUlRyS4AdTrXYfcK3C3p-N9bYE32Mmcnx_UD9jlJ4j_ywqhRPGCs-bOBYMzazzrHJby435bHsqrzXwXpIrZtWWNf_K1bIxWA$)).

Thank you for highlighting this. We agree that the extensive literature describing reproductive dysfunction during *T. vivax* infection provides important context for our findings. We have therefore expanded the Discussion to acknowledge previous studies demonstrating disrupted oestrous cycling, anoestrus, impaired ovarian function and altered progesterone production during *T. vivax* infection in livestock. We have also specifically discussed Rodrigues et al. (2013), as suggested by the reviewer, which demonstrated reduced ovarian size, absence of corpora lutea and abnormal follicular development in experimentally infected goats. Several of these changes closely resemble the reproductive abnormalities we observed during chronic *T. b. brucei* infection.

**Lines:** 514-521.

Very minor/formatting comments:

Fig 1A T.b.b. (dot missing on the last b)

Fig 1E Log10 – 10 should be subscripted

Fig S2C/D and S8 D/E: what is the scale/unit in these panels?

Line 196: Fig 2J should be bold

Fig 5E/F: edit axis name to remove “_” signs, subscript 2 in log2FoldChange and log2(FoldEnrichment), and edit to include spaces between words. (e.g. Log2 Fold Change)

Thank you for spotting these textual and formatting errors. We have modified the manuscript accordingly.

Reviewer #2: - The authors do not mention (or at least this reviewer could not find it) whether females were staged for estrous cycle phase prior to the anatomopathological and immunological analyses. Uterine size, vascularization, and infiltrating immune cell subpopulations vary significantly across estrous cycle phases. Therefore, all analyses should be performed on animals synchronized to the same estrous cycle phase to avoid confounding the results.

Thank you for this comment. The animals were analysed at their naturally occurring oestrous stage rather than being stage matched, although oestrous stage was recorded for each animal at harvest (lines 616-625). We acknowledge that oestrous cycling influences uterine structure and immunology and may therefore contribute to the variation observed within our naive animals. However, oestrous cyclicity was itself a readout of interest in this study and was profoundly disrupted by infection, making stage matching between naive and infected animals impractical. Although we considered oestrous synchronisation with medroxyprogesterone acetate, its known immunomodulatory effects could themselves alter the uterine immune response and natural infection course [3].

Regarding the uterine immune profiling, the reported proportion of infiltrating eosinophils in naïve females (approximately 45% of total leukocytes; Supplementary Figures 4 and 9) is remarkably high. These data sharply contrast with those reported by Diener et al. (J Reprod Immunol 2016; [http://dx.doi.org/10.1016/j.jri.2015.11.005 [dx.doi.org]](https://urldefense.com/v3/__http:/dx.doi.org/10.1016/j.jri.2015.11.005__;!!PDiH4ENfjr2_Jw!G56s9wbUlRyS4AdTrXYfcK3C3p-N9bYE32Mmcnx_UD9jlJ4j_ywqhRPGCs-bOBYMzazzrHJby435bHsqrzXwXpIrZtWWNf_Zd4xOPg$)), who described uterine eosinophil frequencies of 8–20% across the estrous cycle. Which phenotypic markers were used for their identification? Furthermore, if this finding is confirmed, the authors do not discuss why eosinophils are almost completely depleted following parasitic infection at both timepoints (Supplementary Figure 4B).

We thank the reviewer for highlighting this difference. Eosinophils were identified as live CD45^+^SiglecF^+^SSC-high cells, using both the eosinophil-associated SiglecF marker and the characteristic high granularity of eosinophils to distinguish them from other uterine myeloid cells. The gating strategy is described (Sup. Fig. 4) and we have also detailed the markers used in the results (lines 207-219).

We agree that the proportion of eosinophils detected in naive uteri in our study was higher than that reported by Diener et al. Eosinophils are particularly sensitive to tissue digestion and readily incorporate viability dyes, potentially resulting in their exclusion during live-cell gating. An important difference is the methodology used to digest the uterine tissue. Our protocol uses collagenase D and V and Dispase II, whereas the protocol described by Diener et al. did not include these enzymes. As eosinophils are present throughout the uterine stroma, differences in tissue digestion may affect both their release from the tissue and their subsequent viability. Differences in oestrous stage, cell recovery and flow-cytometric analysis may therefore contribute to the different eosinophil frequencies reported between the studies.

We have also expanded the discussion section regarding the reduction in uterine eosinophils during infection. Uterine eosinophils are regulated by the local hormonal and cytokine environment, and both of these are dysregulated during *T. brucei* infection. Their depletion may reflect a disruption to the signals that recruit eosinophils to the uterus during homeostasis. However, we did not perform experiments to determine the mechanism of eosinophil loss.

**Lines:** 433-441.

It is intriguing that most infiltrating monocytes express Ym1 at both stages of infection, consistent with an alternatively activated phenotype, whereas infiltrating macrophages and conventional dendritic cells display a pro-inflammatory phenotype. The authors do not adequately discuss this interesting finding. Could this discrepancy be related to the origin of these cell populations (tissue-resident versus blood-derived)? Please, discuss these data.

We agree that the expression of Ym1 on monocytes is an interesting observation. Although Ym1 has historically been viewed as a marker of alternative activation, it is increasingly recognised as a marker expressed by myeloid cells during tissue remodelling and repair [6]. Ym1 expression may therefore reflect a monocyte phenotype combining inflammatory and tissue-remodelling properties, rather than a strictly alternatively activated phenotype [7]. Given that parasites were detected in the uterus during acute and chronic infection, we speculate that Ym1 expression on monocytes may reflect activation associated with tissue remodelling and repair in response to parasite colonisation.

**Lines:** 441-446.

At the beginning of the Results section (lines 158–159), the authors state that 'Although T. brucei is the most commonly used strain for rodent models, whether this parasite can invade the FRT has not been researched.' However, a previous report assessing a murine model of female reproductive tract infection by T. brucei gambiense is mentioned in the Introduction (lines 90–94). Please correct this discrepancy and, more importantly, clearly state the differences between both studies and highlight the novelty of the present manuscript.

Thank you for this comment. We have updated the text to highlight that *T. brucei brucei* is the most commonly used subspecies, a different subspecies from *T. brucei gambiense.* Whilst the previous study demonstrated that *T. b. gambiense* can invade the FRT using bioluminescent imaging, it did not investigate the structural, endocrinological and immunological consequences of infection within the uterus or ovary. In contrast, our study investigates the effects of *T. b. brucei* infection on the FRT, including changes to the uterine and ovarian morphology, oestrous cycle, uterine immune remodelling, and transcriptional dysregulation across the HPG axis.

**Lines:** 158.

Results from parasite detection and quantification in uterine tissue are contradictory. While immunohistochemistry showed similar parasite burden between the acute and chronic phases of infection, molecular analysis by qRT-PCR revealed the opposite. When discussing this discrepancy, the authors state that animals were not perfused prior to tissue collection, and therefore some of the qPCR-detected Pfr2 signal could have originated from parasites localized within the uterine vasculature rather than the tissue itself. I suggest that the authors either repeat the analysis using properly perfused tissue and present these corrected qRT-PCR results, or rely solely on the unequivocal immunohistochemistry findings for their conclusions.

Thank you for this comment. To address this concern, we have repeated the parasite quantification using perfused chronically infected animals to minimise the contribution of parasites from the vasculature. The additional analysis is displayed (Sup. Fig. 1B), along with additional representative histological sections demonstrating parasite localisation in the uterine tissue (Sup. Fig. 1A). We have updated the methods accordingly.

Ovulation rates should be analyzed and shown in figures 2 and 6.

We thank the reviewer for this suggestion. Whilst we did not directly measure ovulation rates, we quantified the number of corpora lutea relative to the total number of follicles (primary, secondary and antral) as an indirect measure of ovulatory function. These data are now presented in Fig. 4F alongside the number of atretic follicles.

The paragraph at the end of the Results section (lines 388–393) should be more cautious, given that reproductive performance was not assessed and several hormones, including testosterone, prolactin, and anti-Müllerian hormone, were not measured.

Thank you for this comment. We have altered the text throughout to more accurately reflect our findings.

**Lines:** 384-385.

Discussion section, lines 541-545: “A limitation of the current study is that tamoxifen acts through selective modulation of oestrogen receptors and is known to cause uterotrophic effects [95]. Therefore, it is unclear whether restoration of uterine morphology reflects restoration of the infection-induced endocrine dysfunction or direct mitogenic activity of tamoxifen in the uterus”. What about the effects on the restoration of estrous cycling? This should also be addressed, as it would help distinguish between a genuine recovery of endocrine/ovarian function versus a direct, cycle-independent uterotrophic effect of tamoxifen.

We thank the reviewer for this insightful comment and agree that the difference in oestrous cyclicity warrants further discussion. We have revised both the results and discussion to avoid overinterpreting the oestrous findings. We now additionally acknowledge that, whilst tamoxifen administration prevented infection-induced accumulation within proestrus, it is not clear whether this is genuine restoration of the HPG axis or a pharmacological effect of tamoxifen.

**Lines:** 544-547, 558-561.

Discussion section, lines 545-546: “An additional limitation is that circulating oestradiol concentrations were measured by ELISA [96,97]”. Why is that a limitation?

Thank you for highlighting this. We have revised the discussion to clarify why measurement of oestradiol by ELISA is a limitation. Since oestradiol circulates at very low concentrations in mice, accurate quantification by ELISA is challenging due to assay sensitivity and specificity. Therefore, confirming these findings using a more sensitive analytical method would be important in future studies.

**Lines:** 562-567.
Discussion section, lines 569-570: “Collectively, our findings identify the female reproductive system as a major target of systemic inflammatory remodeling during T. brucei infection”. The authors analyzed local rather than systemic immune changes. Please rephrase this statement accordingly.

Thank you for highlighting this. We have revised the text to state that the local uterine immune landscape is remodelled in response to *T. brucei* infection, which more accurately reflects the data presented within the manuscript.

**Lines:** 595-596.

**References:**

1.	Walker MK, Boberg JR, Walsh MT, Wolf V, Trujillo A, Duke MS, et al. A less stressful alternative to oral gavage for pharmacological and toxicological studies in mice. Toxicol Appl Pharmacol. 2012;260: 65–69. doi:10.1016/j.taap.2012.01.025

2.	Vanhecke D, Bugada V, Steiner R, Polić B, Buch T. Refined tamoxifen administration in mice by encouraging voluntary consumption of palatable formulations. Lab Anim. 2024;53: 205–214. doi:10.1038/s41684-024-01409-z

3.	Kleynhans L, Du Plessis N, Allie N, Jacobs M, Kidd M, van Helden PD, et al. The Contraceptive Depot Medroxyprogesterone Acetate Impairs Mycobacterial Control and Inhibits Cytokine Secretion in Mice Infected with Mycobacterium tuberculosis. Infect Immun. 2013;81: 1234–1244. doi:10.1128/IAI.01189-12

4.	Silva LAS, Felix FB, Araujo JMD, Souza EV, Camargo EA, Grespan R. Agonistic activity of tamoxifen, a selective estrogen-receptor modulator (SERM), on arthritic ovariectomized mice. Braz J Med Biol Res. 2017;51: e6799. doi:10.1590/1414-431X20176799

5.	Sinton MC, Chandrasegaran PRG, Capewell P, Cooper A, Girard A, Ogunsola J, et al. IL-17 signalling is critical for controlling subcutaneous adipose tissue dynamics and parasite burden during chronic murine Trypanosoma brucei infection. Nat Commun. 2023;14: 7070. doi:10.1038/s41467-023-42918-8

6.	Kang Q, Li L, Pang Y, Zhu W, Meng L. An update on Ym1 and its immunoregulatory role in diseases. Front Immunol. 2022;13: 891220. doi:10.3389/fimmu.2022.891220

7.	Scott NA, Mohiyiddeen L, Mariano LL, Ruane PT, Aplin JD, Moran OC, et al. Macrophages in the uterus are functionally specialised and continually replenished from the circulation. bioRxiv; 2021. p. 2021.09.02.458731. doi:10.1101/2021.09.02.458731
