# Supplementary material for: Trypanosoma brucei infection remodels the uterine immune environment and drives neuroendocrine dysfunction
Source: PLoS Pathog. 2026 Oct 9;22(10):e1014667. doi: 10.1371/journal.ppat.1014667 (PMC13671655; doi:10.1371/journal.ppat.1014667)
Supplement: Peer review history

# Peer review: Decision Letter 0

**DOI:** 10.1371/journal.ppat.1014667.r001

**Authors:** Margaret Phillips, Álvaro Acosta-Serrano

15 Jul 2026

PPATHOGENS-D-26-01488

Trypanosoma brucei  infection remodels the uterine immune environment and drives neuroendocrine dysfunction

PLOS Pathogens

Dear Dr. Quintana,

Thank you for submitting your manuscript to PLOS Pathogens. After careful consideration, we feel that it has merit but does not fully meet PLOS Pathogens's publication criteria as it currently stands. Therefore, we invite you to submit a revised version of the manuscript that addresses the points raised during the review process. **Note from the Editor:**  As you will see, both reviewers are generally supportive of the work, but they have raised several concerns that should be carefully addressed in a revised manuscript.

Please submit your revised manuscript by Sep 13 2026 11:59PM. If you will need more time than this to complete your revisions, please reply to this message or contact the journal office at plospathogens@plos.org. When you're ready to submit your revision, log on to https://www.editorialmanager.com/ppathogens/ and select the 'Submissions Needing Revision' folder to locate your manuscript file.

Please include the following items when submitting your revised manuscript:

* A letter that responds to each point raised by the editor and reviewer(s). You should upload this letter as a separate file labeled 'Response to Reviewers'. This file does not need to include responses to any formatting updates and technical items listed in the 'Journal Requirements' section below.

* A marked-up copy of your manuscript that highlights changes made to the original version. You should upload this as a separate file labeled 'Revised Manuscript with Track Changes'.

* An unmarked version of your revised paper without tracked changes. You should upload this as a separate file labeled 'Manuscript'.

If you would like to make changes to your financial disclosure, competing interests statement, or data availability statement, please make these updates within the submission form at the time of resubmission. Guidelines for resubmitting your figure files are available below the reviewer comments at the end of this letter. As the corresponding author, your ORCID iD is verified in the submission system and will appear in the published article. PLOS supports the use of ORCID, and we encourage all coauthors to register for an ORCID iD and use it as well. Please encourage your coauthors to verify their ORCID iD within the submission system before final acceptance, as unverified ORCID iDs will not appear in the published article. Only the individual author can complete the verification step; PLOS staff cannot verify ORCID iDs on behalf of authors.

We look forward to receiving your revised manuscript.

Kind regards,

Álvaro Acosta-Serrano

Academic Editor

PLOS Pathogens

Margaret Phillips

Section Editor

PLOS PathogensSumita Bhaduri-McIntosh

Editor-in-Chief

PLOS Pathogens

orcid.org/0000-0003-2946-9497

Michael Malim

Editor-in-Chief

PLOS Pathogens

orcid.org/0000-0002-7699-2064

**Additional Editor Comments (if provided):**

**Journal Requirements:**

If the reviewer comments include a recommendation to cite specific previously published works, please review and evaluate these publications to determine whether they are relevant and should be cited. There is no requirement to cite these works unless the editor has indicated otherwise.

1) Please ensure that the CRediT author contributions listed for every co-author are completed accurately and in full.

At this stage, the following Authors/Authors require contributions: Olivia M Shorthouse, Chloe Barnes, Stefano Colombo, Jessica Costa, Katrine Wonsbek, Anmoyul Mohon, Andrew S MacDonald, Elizabeth Mann, Alice H Costain, and Juan F. Quintana. Please ensure that the full contributions of each author are acknowledged in the "Add/Edit/Remove Authors" section of our submission form.

The list of CRediT author contributions may be found here: https://journals.plos.org/plospathogens/s/authorship#loc-author-contributions

2) We do not publish any copyright or trademark symbols that usually accompany proprietary names, eg ©,  ®, or TM  (e.g. next to drug or reagent names). Therefore please remove all instances of trademark/copyright symbols throughout the text, including:

- ® on page: 26

- TM on page: 15.

3) Please upload all main figures as separate Figure files in .tif or .eps format. For more information about how to convert and format your figure files please see our guidelines:

https://journals.plos.org/plospathogens/s/figures

4) Please amend your detailed Financial Disclosure statement. This is published with the article. It must therefore be completed in full sentences and contain the exact wording you wish to be published.

i) State what role the funders took in the study. If the funders had no role in your study, please state: "The funders had no role in study design, data collection and analysis, decision to publish, or preparation of the manuscript."

ii) If any authors received a salary from any of your funders, please state which authors and which funders..

If you did not receive any funding for this study, please simply state: u201cThe authors received no specific funding for this work.u201d

5) We have amended your Competing Interest statement to comply with journal style. We kindly ask that you double check the statement and let us know if anything is incorrect.

6) In the online submission form, you indicated that your data will be submitted to a repository upon acceptance. We strongly recommend all authors deposit their data before acceptance, as the process can be lengthy and hold up publication timelines. Please note that, though access restrictions are acceptable now, your entire minimal dataset will need to be made freely accessible if your manuscript is accepted for publication. This policy applies to all data except where public deposition would breach compliance with the protocol approved by your research ethics board. If you are unable to adhere to our open data policy, please kindly revise your statement to explain your reasoning and we will seek the editor's input on an exemption.

**Reviewers' Comments:**

Reviewer's Responses to Questions

**Part I - Summary**

Please use this section to discuss strengths/weaknesses of study, novelty/significance, general execution and scholarship.

Reviewer #1: Shorthouse et al describe how T. brucei brucei remodels the female reproductive tract of infected mice and the associated changes in immune landscape. The manuscript is well-written, experimentally-sound and worth it of publication in this journal, if some concerns are adequately addressed.

Reviewer #2: In the present manuscript, Shorthouse et al. provide data from the analysis of the female reproductive tract and local immune and endocrine biomarkers in a mouse model of Trypanosoma brucei brucei infection in C57BL/6 mice. Authors present a considerable amount of original data showing the presence of the parasite in the endometrium in the acute as well as the chronic phases of the infection. Moreover, chronic infection was associated with decreased body fat tissue, increased estradiol and decreased progesterone levels, altered estrous cycling, considerable atrophy of the uterus and ovaries, and transcriptional dysegulation of the hypothalamic-pituitary-gonadal (HPG) axis. Interestingly, authors found that these changes were accompanied by a profound remodeling of the uterine immune milieu, characterized by the increased infiltration of leukocytes (only in the chronic phase of the infection) and higher proportion of infiltrating T cells (either in the acute or in the chronic phase of the infection), and heightened expression of IFN-gamma, showing a biased Type 1 response. Interestingly, the treatment with tamoxifen, an estrogen receptor agonist within the uterus and ovaries, reversed uterine pathology and rescued estrous cycle dynamics but did not reverse uterine immune remodeling. Based on these findings, authors conclude that the female reproductive tract is a major target of systemic T. brucei infection, which consequently induces uterine and ovarian pathology, HPG transcriptional dysregulation, and uterine immune dysregulation despite hormonal rescue. Based on their findings, authors highlight the need to incorporate female reproductive health into studies of systemic infection and neglected tropical diseases.

The work addresses a very important and neglected clinical condition: reproductive dysfunction in African trypanosomiasis. The manuscript is well-written, focused, and easy to follow, and the results presented are novel, overall compelling, and make a significant contribution to the field, filling an existing knowledge gap. However, the work has some weaknesses that could be addressed through moderate revision, which would further support the conclusions drawn and strengthen the overall quality of the manuscript.

**********

**Part II – Major Issues: Key Experiments Required for Acceptance**

Please use this section to detail the key new experiments or modifications of existing experiments that should be absolutely required to validate study conclusions.

Generally, there should be no more than 3 such required experiments or major modifications for a "Major Revision" recommendation. If more than 3 experiments are necessary to validate the study conclusions, then you are encouraged to recommend "Reject".

Reviewer #1: 1. Sup Fig 8B There is considerable variation in the Pfr2 copies/mg tissue in non-tamoxifen treated infected animals, which might be skewing the statistical analysis. I would recommend increasing the N of this experiment to confirm its findings.

2. Lines 416-418: the authors acknowledge that the mice were not perfused prior to tissue collection. This is a red line when discussing tissue tropism. Based on previous literature reporting tissue load vs. parasitaemia, the lack of perfusion will considerably overestimate parasite burden. Based on this point and the previous one, I suggest that claims of tropism to the FRT to be toned down or supported with actual parasite quantification in the FRT extravascular spaces. Currently, only a representative H&E stain is provided. Considering this and the high variation in the Pfr2 RT-qPCR (Sup Fig 8B), it is my recommendation that an experiment is conducted to assess parasite burden in perfused FRT.

3. Line 347: Why was tamoxifen administered by voluntary feeding rather than standard injection and how can the authors be sure the amount of tamoxifen ingested was similar between naïve and infected groups? I think this is particularly relevant given that T brucei infection is often associated with periods of anorexia where food intake is significantly reduced.

Reviewer #2: Major concerns:

- The characterization of uterine immune cell infiltration is presented in an unclear and unconventional manner. The authors should present total counts and frequencies of CD45+ leukocytes, together with all subpopulations, within the same figure (Figures 3 and 7). To clearly demonstrate the claimed differences, total CD45+ leukocyte counts and the frequencies of myeloid and lymphoid subsets (eosinophils, neutrophils, monocytes, dendritic cells, B cells, and T cells) should be consolidated into a single figure. Moreover, it is puzzling that the authors do not report NK cell data, given their well-established importance in uterine physiology and reproduction. In which stage of the estrous cycle were these analyses performed? Besides, the combination of markers used for the identification of each leukocyte subset by flow cytometry is not clearly detailed in the manuscript.

- The authors hypothesize that uterine and ovarian atrophy (which co-occurred with increased serum estradiol levels alongside significantly reduced progesterone levels) resulted from impaired uterine responsiveness to endogenous estrogens. Consequently, they tested the efficacy of tamoxifen, an estrogen receptor agonist in the uterus and ovaries, in reversing the observed changes. Although interesting, this hypothesis is rather peculiar given that systemic estrogen levels were already elevated while progesterone levels were significantly reduced. Furthermore, transcriptional analyses revealed unchanged Esr1 expression in the ovary. Would it not have been more rational to hypothesize defects resulting from progesterone deficiency and, accordingly, test progesterone replacement therapy? In that regard, authors sustain that tamoxifen treatment reverted uterine pathology and estrous cycling. Were those analyses performed in females staged for estrous cycle phase?

- Although interesting, the presented data do not clearly demonstrate that the infection decreased reproductive potential. Mating experiments assessing potency, fecundity, and fertility index, as well as rates of pre- and post-implantation embryo loss, would significantly strengthen the quality of the manuscript.

**********

**Part III – Minor Issues: Editorial and Data Presentation Modifications**

Please use this section for editorial suggestions as well as relatively minor modifications of existing data that would enhance clarity.

Reviewer #1: 1. Lines 190-193: Despite the common splenomegaly and hepatomegaly, weight loss is usually reported during T. b. b. infection. Could the authors speculate on why that was not observed here? I think it is worth noting in the text this discrepancy with current literature. A few thoughts:

- Was there evidence for fluid accumulation in the lungs of infected mice that could also contribute to the unchanged body weight?

- Could female be more resistant to cachexia/muscle wasting than males (given that the majority of reports have used male mice)?

2. Lines 202-204: Can the authors explain what is the difference between the frequency of CD45+ cells (changed) and the total numbers of CD45+ cells / mg tissue (unchanged)? How is the frequency calculated and what could explain this discrepancy? Is it linked to the infection-induced uterine atrophy? If so, is there a true expansion/recruitment of leukocytes to the FRT if the absolute numbers of cells remain unchanged? And how does total number of cells remain unchanged if there is a clear expansion of CD3+ cells (described in subsequent sentences)?

3. Lines 220-223: There is no significant increase in CD4+ T cells during acute infection. I suggest the sentence should be improved for clarity. Moreover, would it be possible to show the full composition of the immune repertoire rather than just CD4 and CD8+ T cells? Assuming the authors already have these data (alluded, for example, in the no. of macrophages and neutrophils), it would be helpful to have a weighted bar chart in naïve, acute and chronic with all CD45+ cells and the proportion of each cell subset assessed.

4. Still in the previous line, why were only chronically infected animal subjected to this treatment? Is there a reason to not assess acute infection? Given that all other figures so far have included both stages of infection, it would make sense to include it here too.

5. Fig6J and lines 362-365: the cycle profiles with tamoxifen do not reproduce a naïve animal. It would perhaps be more accurate to state that “during infection, and despite altered oestrous cycle, the HPG axis partially retains the capacity to respond to oestrogenic signals”.

6. Throughout the intro and results sections and very clearly on the concluding sentence of the results sections (lines 392-3), it appears that the authors would expect immune remodeling to be driven by hormonal disruption. Is this the case? If so, why would it be expected? If not, then I’d suggest rephrasing.

7. Lines 411-413: I think it is overall agreed in the field that T. brucei can infect and establish in all organs. This has been reported in the early studies by Ikede, Losos and others in the 70s, but also recently by, for example, De Niz et al 2021. So, one could argue that the FRT does not come as an “unappreciated site of … tropism”, but it is rather an organ that, like all others observed to date, does not offer any effective barrier for parasite invasion. Whether we consider tropism as the ability of a microenvironment to support infection, or a preference of the microorganism for a given microenvironment is debatable, but I have seen no evidence in this paper to suggest that the FRT is a preferential site of parasite colonization.

8. Lines 626-639: Following the point above, the title and description of this methods section should be corrected to acknowledge that what is measured is the total parasite burden in the uterus and associated vasculature, and not the tissue parasite burden.

9. Lines 424-427: The authors mention that congenital transmission has been reported in both experimental models and humans, but cite papers 58, 59, and 60. Ref 58 and 60 are two human reports, ref 59 is a short review that does not (to my knowledge) include any reference to experimental models. Is there a reference missing? I think it would also be important to acknowledge how many cases have been reported, because it appears to be less than 20 cases reported in almost a century (1933?). So, whilst I don’t argue that it might be underreported, I also consider that, if it were relatively frequent, sleeping sickness with such a severe clinical presentation would be hard to miss.

10. Lines 427-429: Have the authors tried to breed infected mice to assess evidence for congenital transmission?

11. Still on the topic of congenital transmission and FRT remodeling, and especially given that the paper uses T. brucei brucei, an agent of nagana and not sleeping sickness, I am surprised papers describing T vivax-induced FRT changes are not cited nor discussed. I think discussing previous work on how T vivax affects the FRT (in a natural host) would strengthen the discussion by establishing precedent (ref: https://doi.org/10.1016/j.vetpar.2012.08.001).

Very minor/formatting comments:

Fig 1A T.b.b. (dot missing on the last b)

Fig 1E Log10 – 10 should be subscripted

Fig S2C/D and S8 D/E: what is the scale/unit in these panels?

Line 196: Fig 2J should be bold

Fig 5E/F: edit axis name to remove “_” signs, subscript 2 in log2FoldChange and log2(FoldEnrichment), and edit to include spaces between words. (e.g. Log2 Fold Change)

Reviewer #2: - The authors do not mention (or at least this reviewer could not find it) whether females were staged for estrous cycle phase prior to the anatomopathological and immunological analyses. Uterine size, vascularization, and infiltrating immune cell subpopulations vary significantly across estrous cycle phases. Therefore, all analyses should be performed on animals synchronized to the same estrous cycle phase to avoid confounding the results.

- Regarding the uterine immune profiling, the reported proportion of infiltrating eosinophils in naïve females (approximately 45% of total leukocytes; Supplementary Figures 4 and 9) is remarkably high. These data sharply contrast with those reported by Diener et al. (J Reprod Immunol 2016; http://dx.doi.org/10.1016/j.jri.2015.11.005), who described uterine eosinophil frequencies of 8–20% across the estrous cycle. Which phenotypic markers were used for their identification? Furthermore, if this finding is confirmed, the authors do not discuss why eosinophils are almost completely depleted following parasitic infection at both timepoints (Supplementary Figure 4B).

- It is intriguing that most infiltrating monocytes express Ym1 at both stages of infection, consistent with an alternatively activated phenotype, whereas infiltrating macrophages and conventional dendritic cells display a pro-inflammatory phenotype. The authors do not adequately discuss this interesting finding. Could this discrepancy be related to the origin of these cell populations (tissue-resident versus blood-derived)? Please, discuss these data.

- At the beginning of the Results section (lines 158–159), the authors state that 'Although T. brucei is the most commonly used strain for rodent models, whether this parasite can invade the FRT has not been researched.' However, a previous report assessing a murine model of female reproductive tract infection by T. brucei gambiense is mentioned in the Introduction (lines 90–94). Please correct this discrepancy and, more importantly, clearly state the differences between both studies and highlight the novelty of the present manuscript.

- Results from parasite detection and quantification in uterine tissue are contradictory. While immunohistochemistry showed similar parasite burden between the acute and chronic phases of infection, molecular analysis by qRT-PCR revealed the opposite. When discussing this discrepancy, the authors state that animals were not perfused prior to tissue collection, and therefore some of the qPCR-detected Pfr2 signal could have originated from parasites localized within the uterine vasculature rather than the tissue itself. I suggest that the authors either repeat the analysis using properly perfused tissue and present these corrected qRT-PCR results, or rely solely on the unequivocal immunohistochemistry findings for their conclusions.

- Ovulation rates should be analyzed and shown in figures 2 and 6.

- The paragraph at the end of the Results section (lines 388–393) should be more cautious, given that reproductive performance was not assessed and several hormones, including testosterone, prolactin, and anti-Müllerian hormone, were not measured.

- Discussion section, lines 541-545: “A limitation of the current study is that tamoxifen acts through selective modulation of oestrogen receptors and is known to cause uterotrophic effects [95]. Therefore, it is unclear whether restoration of uterine morphology reflects restoration of the infection-induced endocrine dysfunction or direct mitogenic activity of tamoxifen in the uterus”. What about the effects on the restoration of estrous cycling? This should also be addressed, as it would help distinguish between a genuine recovery of endocrine/ovarian function versus a direct, cycle-independent uterotrophic effect of tamoxifen.

- Discussion section, lines 545-546: “An additional limitation is that circulating oestradiol concentrations were measured by ELISA [96,97]”. Why is that a limitation?

- Discussion section, lines 569-570: “Collectively, our findings identify the female reproductive system as a major target of systemic inflammatory remodeling during T. brucei infection”. The authors analyzed local rather than systemic immune changes. Please rephrase this statement accordingly.

**********

PLOS authors have the option to publish the peer review history of their article (what does this mean?). If published, this will include your full peer review and any attached files.

If you choose “no”, your identity will remain anonymous but your review may still be made public.

**Do you want your identity to be public for this peer review?**  For information about this choice, including consent withdrawal, please see our Privacy Policy.

Reviewer #1: No

Reviewer #2: No

[NOTE: If reviewer comments were submitted as an attachment file, they will be attached to this email and accessible via the submission site. Please log into your account, locate the manuscript record, and check for the action link "View Attachments". If this link does not appear, there are no attachment files.]

**Figure resubmission:**

While revising your submission, we strongly recommend that you use PLOS’s NAAS tool (https://ngplosjournals.pagemajik.ai/artanalysis) to test your figure files. NAAS can convert your figure files to the TIFF file type and meet basic requirements (such as print size, resolution), or provide you with a report on issues that do not meet our requirements and that NAAS cannot fix.

After uploading your figures to PLOS’s NAAS tool - https://ngplosjournals.pagemajik.ai/artanalysis, NAAS will process the files provided and display the results in the "Uploaded Files" section of the page as the processing is complete. If the uploaded figures meet our requirements (or NAAS is able to fix the files to meet our requirements), the figure will be marked as "fixed" above. If NAAS is unable to fix the files, a red "failed" label will appear above. When NAAS has confirmed that the figure files meet our requirements, please download the file via the download option, and include these NAAS processed figure files when submitting your revised manuscript.

**Reproducibility:**

To enhance the reproducibility of your results, we recommend that authors of applicable studies deposit laboratory protocols in protocols.io, where a protocol can be assigned its own identifier (DOI) such that it can be cited independently in the future. Additionally, PLOS ONE offers an option to publish peer-reviewed clinical study protocols. Read more information on sharing protocols at https://plos.org/protocols?utm_medium=editorial-email&utm_source=authorletters&utm_campaign=protocols

# Author comment: Author response to Decision Letter 1

**DOI:** 10.1371/journal.ppat.1014667.r002

10 Sep 2026

Attachment Submitted filename: Response to reviewers_020926.docx

# Peer review: Decision Letter 1

**DOI:** 10.1371/journal.ppat.1014667.r003

**Authors:** Margaret Phillips, Álvaro Acosta-Serrano

25 Sep 2026

Dear Dr. Quintana,

We are pleased to inform that your manuscript, "Trypanosoma brucei infection remodels the uterine immune environment and drives neuroendocrine dysfunction", has been editorially accepted for publication at PLOS Pathogens.

Before your manuscript can be formally accepted and sent to production, you will need to complete our formatting changes, which you will receive by email within a week. Please note that your manuscript will not be scheduled for publication until you have made the required changes.

Note from the Editor: Please address the minor corrections suggested by Reviewer #1 before submitting the final version of the manuscript.

IMPORTANT NOTES

(1) Copyediting and Proofreading: The corresponding author will receive a typeset proof for review, to ensure errors have not been introduced during production. Please review the PDF proof of your manuscript carefully, as this is the last chance to correct any errors. Please note that major changes, or those which affect the scientific understanding of the work, will likely cause delays to the publication date of your manuscript.

(2) Appropriate Figure Files: Please remove all name and figure # text from your figure files. Please also take this time to check that your figures are of high resolution, which will improve the readbility of your figures and help expedite your manuscript's publication. Please note that figures must have been originally created at 300dpi or higher. Do not manually increase the resolution of your files. For instructions on how to properly obtain high quality images, please review our Figure Guidelines, with examples at: http://journals.plos.org/plospathogens/s/figures.

We encourage you to use PACE (http://pace.apexcovantage.com/), a figure-checking tool which helps authors ensure all figures meet PLOS requirements so that figures can publish in the highest quality. PACE is a digital diagnostic and conversion tool for figure files. It will provide information about any failed check(s) and, if able, will automatically convert the figure into an acceptable file that passes quality checks.

To use PACE, you must first register as a user. Then, login and navigate to the UPLOAD tab, where you will find detailed instructions on how to use the tool.

(3) Striking Image: Please upload a striking still image to accompany your article if one is available (you can include a new image or an existing one from within your manuscript). Should your paper be accepted, this image will be considered for our monthly issue image and may also appear on our website to feature your article. Please upload this as a separate file, selecting "striking image" as the file type upon upload. Please also include a separate "Other" file with a caption, including credits and any potential copyright information. Please do not include the caption in the main article file. If your image is from someone other than yourself, please ensure that the artist has read and agreed to the terms and conditions of the Creative Commons Attribution License at http://journals.plos.org/plospathogens/s/content-license. Please note that PLOS cannot publish copyrighted images.

(4) Press Release or Related Media: If you or your institution will be preparing press materials for this manuscript, please inform our press team in advance at plospathogens@plos.org as soon as possible. We ask that you contact us within one week to plan ahead of our fast Production schedule. If you need to know your paper's publication date for related media purposes, you must coordinate with our press team, and your manuscript will remain under a strict press embargo until the publication date and time.

(5) Update your Profile Information: Now that your manuscript has been provisionally accepted, please log into Editorial Manager and update your profile, if needed. Go to http://www.editorialmanager.com/ppathogens, log in, and click on the "Update My Information" link at the top of the page. Please update your user information to ensure an efficient production and billing process.

(6) LaTeX users only: Our staff will ask you to upload a TEX file in addition to the PDF before the paper can be sent to typesetting, so please carefully review our Latex Guidelines http://journals.plos.org/plospathogens/s/latex in the meantime.

Thank you again for supporting Open Access publishing; we are looking forward to publishing your work in PLOS Pathogens.

Best regards,

Álvaro Acosta-Serrano

Academic Editor

PLOS Pathogens

Margaret Phillips

Section Editor

PLOS Pathogens

Dominique Soldati-Favre

Editor-in-Chief

PLOS Pathogens

***********************************************************

Reviewer's Responses to Questions

**Part I - Summary**

Please use this section to discuss strengths/weaknesses of study, novelty/significance, general execution and scholarship.<br/><br/>

Reviewer #1: I thank the authors for responding to my comments and addressing some of the limitations identified.

I am happy to endorse publication of this article, as long as the minor issues are corrected.

Reviewer #2: (No Response)

--------------------

**Part II – Major Issues: Key Experiments Required for Acceptance**

Please use this section to detail the key new experiments or modifications of existing experiments that should be absolutely required to validate study conclusions.<br/><br/>Generally, there should be no more than 3 such required experiments or major modifications for a "Major Revision" recommendation. If more than 3 experiments are necessary to validate the study conclusions, then you are encouraged to recommend "Reject".<br/><br/>

Reviewer #1: Nothing to add.

Reviewer #2: In the present manuscript, Shorthouse et al. provide data from the analysis of the female reproductive tract and local immune and endocrine biomarkers in a mouse model of Trypanosoma brucei brucei infection in C57BL/6 mice. Authors present a considerable amount of original data showing the presence of the parasite in the endometrium in the acute as well as the chronic phases of the infection. Moreover, chronic infection was associated with decreased body fat tissue, increased estradiol and decreased progesterone levels, altered estrous cycling, considerable atrophy of the uterus and ovaries, and transcriptional dysegulation of the hypothalamic-pituitary-gonadal (HPG) axis. Interestingly, authors found that these changes were accompanied by a profound remodeling of the uterine immune milieu, characterized by the increased infiltration of leukocytes (only in the chronic phase of the infection) and higher proportion of infiltrating T cells (either in the acute or in the chronic phase of the infection), and heightened expression of IFN-gamma, showing a biased Type 1 response. Interestingly, the treatment with tamoxifen, an estrogen receptor agonist within the uterus and ovaries, reversed uterine pathology and rescued estrous cycle dynamics but did not reverse uterine immune remodeling. Based on these findings, authors conclude that the female reproductive tract is a major target of systemic T. brucei infection, which consequently induces uterine and ovarian pathology, HPG transcriptional dysregulation, and uterine immune dysregulation despite hormonal rescue. Consequently, authors highlight the need to incorporate female reproductive health into studies of systemic infection and neglected tropical diseases.

The work addresses a very important and neglected clinical condition: reproductive dysfunction in African trypanosomiasis. The revised version of the manuscript is well-written, focused, and easy to follow, and the results presented are novel, compelling, and make a significant contribution to the field, filling an existing knowledge gap. Authors have properly addressed most of this reviewers concerns; therefore, the quality of the work has improved significantly. Thus, I recommend its publication.

--------------------

**Part III – Minor Issues: Editorial and Data Presentation Modifications**

Please use this section for editorial suggestions as well as relatively minor modifications of existing data that would enhance clarity.<br/><br/>

Reviewer #1: 1) Line 167: is there a word missing at the end? “Histological analysis using staining for the T. brucei-specific protein BiP showed no detectable staining in naive uterine tissue, whereas parasites were readily detected in the uterus during (Figs 1B-1D).”

2) Fig S1: is BiP stained in brown? It is not mentioned in the figure legend.

3) Fig S1: There's a strange line in the 4th image of panel A. Maybe a scale bar from the original figure? this should be corrected.

Reviewer #2: Authors have properly addressed most of this reviewers concerns; therefore, the quality of the work has improved significantly.

--------------------

PLOS authors have the option to publish the peer review history of their article (what does this mean?). If published, this will include your full peer review and any attached files.

If you choose “no”, your identity will remain anonymous but your review may still be made public.

**Do you want your identity to be public for this peer review?**  For information about this choice, including consent withdrawal, please see our Privacy Policy.

Reviewer #1: Yes: Sara Silva Pereira

Reviewer #2: Yes: Ruben Dario Motrich

# Editor report: Acceptance letter

**DOI:** 10.1371/journal.ppat.1014667.r004

**Authors:** Margaret Phillips, Álvaro Acosta-Serrano

Dear Dr. Quintana,

We are delighted to inform you that your manuscript, "Trypanosoma brucei  infection remodels the uterine immune environment and drives neuroendocrine dysfunction," has been formally accepted for publication in PLOS Pathogens.

We have now passed your article onto the PLOS Production Department who will complete the rest of the pre-publication process. All authors will receive a confirmation email upon publication.

Thank you again for supporting open-access publishing; we are looking forward to publishing your work in PLOS Pathogens.

Best regards,

Dominique Soldati-Favre

Editor-in-Chief

PLOS Pathogens
